# Role of NF-kappaB2-p100 in regulatory T cell homeostasis and activation

**DOI:** 10.1038/s41598-019-50454-z

**Published:** 2019-09-25

**Authors:** Atika Dhar, Meenakshi Chawla, Somdeb Chattopadhyay, Neelam Oswal, Danish Umar, Suman Gupta, Vineeta Bal, Satyajit Rath, Anna George, G. Aneeshkumar Arimbasseri, Soumen Basak

**Affiliations:** National Institute of Immunology, New Delhi, India

**Keywords:** Regulatory T cells, NF-kappaB

## Abstract

The immunological roles of the nuclear factor-kappaB (NF-κB) pathway are mediated via the canonical components in immune responses and via non-canonical components in immune organogenesis and homeostasis, although the two components are capable of crosstalk. Regulatory CD4 T cells (Tregs) are homeostatically functional and represent an interesting potential meeting point of these two NF-κB components. We show that mice deficient in the non-canonical NF-κB component gene *Nfkb2* (p100) had normal thymic development and suppressive function of Tregs. However, they had enhanced frequencies of peripheral ‘effector-phenotype’ Tregs (eTregs). In bi-parental chimeras of wild-type (WT) and *Nfkb2*−/− mice, the *Nfkb2*−/− genotype was over-represented in Tregs, with a further increase in the relative prominence of eTregs. Consistent with distinct properties of eTregs, the *Nfkb2*−/− genotype was more prominent in Tregs in extra-lymphoid tissues such as liver in the bi-parental chimeras. The *Nfkb2*−/− Tregs also displayed greater survival, activation and proliferation *in vivo*. These *Nfkb2*−/− Tregs showed higher nuclear NF-κB activity mainly comprising of RelB-containing dimers, in contrast to the prominence of cRel- and RelA-containing dimers in WT Tregs. Since p100 is an inhibitor of RelB activation as well as a participant as cleaved p52 in RelB nuclear activity, we tested bi-parental chimeras of WT and *Relb*−/− mice, and found normal frequencies of *Relb*−/− Tregs and eTregs in these chimeric mice. Our findings confirm and extend recent data, and indicate that p100 normally restrains RelB-mediated Treg activation, and in the absence of p100, p50-RelB dimers can contribute to Treg activation.

## Introduction

Regulatory T cells expressing the characteristic lineage determining transcription factor FOXP3 are actively involved in suppressing overt activation of the immune system and the development of autoimmunity^1^. Tregs are characterised by constitutive expression of IL-2Rα (CD25)^2^ and of several other molecules that contribute to their suppressive function such as CTLA-4^3^, ICOS^4^ and CD73^5^. The peripheral Treg population includes thymic-origin Tregs (‘natural’ Tregs (nTregs)) and Tregs that have been induced in the periphery from conventional CD4 T cells (‘induced’ Tregs (iTregs))^6^. Tregs perform their functions through a number of mechanisms such as by competing for IL-2 with activated conventional T cells^7^,

by secreting cytokines such as IL-10 which regulates T cell activation and cytokine production^8^, through direct killing mediated by perforin or granzyme production^9^, and by interfering with T cell-dendritic cell interactions^10^.

Tregs in the periphery exhibit heterogeneity and can be categorized into ‘naive’ and ‘effector’ subsets like conventional T cells^11^. Most Tregs in secondary lymphoid organs express high levels of CD62L^12^ and CCR7^13^ like naive conventional T cells, and are termed central Tregs (cTregs)^14^. These cTregs are activated in the periphery^11^ and give rise to effector Tregs (eTregs) showing surface phenotypes similar to recently activated conventional T cells, such as lower levels of CCR7 and CD62L^14^. The eTregs are more prominent in non-lymphoid tissues^13^, short lived^11^, express lower levels of Bcl-2^14^ and contain a greater proportion of homeostatically proliferating cells compared to cTregs^11^. Additionally, eTregs also express higher levels of CD44, GITR, ICOS^11,15^ and chemokine receptors such as CCR4^16^, CCR9^17^, CCR6^18^, etc which direct their migration to specific nonlymphoid tissues.

The cytokine and signaling requirements for maintenance also differ between cTregs and eTregs; while the former depend on IL-2 mediated survival signals, the latter require signaling through ICOS^14^. In addition, sustained T cell receptor (TCR)-mediated signals are also essential for eTreg generation and maintenance. The absence of TCR signals leads to loss of expression of target genes of NFAT indicating its possible role in eTreg generation and maintenance^19,20^. One such NFAT target, IRF4, is indispensable for eTreg generation as well as their trafficking. IRF4 in turn regulates the expression of Blimp1, which is indispensable for the production IL-10 by eTregs and regulates their migration to lymphoid organs as well as non-lymphoid tissues^20,21^.

In addition to IRF4 and BLIMP1, the transcription factors of the canonical NF-κB family have been shown to play a role in eTreg generation and/or maintenance. The NF-κB family of dimeric transcription factors consists of NF-κB1 (p105/p50), NF-κB2 (p100/p52), cRel, RelA (p65) and RelB^22^. The canonical NF-κB pathway, activated downstream of TCR, the tumor necrosis factor receptor (TNFR) and toll like receptor (TLR)-interleukin (IL)-1R superfamilies among others, triggers nuclear translocation of primarily RelA:p50 and cRel:p50 heterodimers by inducing the degradation of IkBα, IkBβ and IkBε^22,23^. *Ex vivo* Tregs, unlike conventional T cells, show p50:RelA and p50:p50 activity under homeostatic conditions and RelA has been shown to regulate eTreg homeostasis^24^. In addition to RelA, cRel also plays a role in activated Treg (aTreg) generation and/or maintenance^25^. Hetero-dimers of p52 and RelB constitute the activity of the non-canonical NF-κB pathway, which is activated through a select set of TNFR superfamily members like CD40, OX-40, GITR and LT-βR^26,27^.

Previous studies have also suggested a role for the noncanonical NF-κB pathway in Treg development and maintenance. The kinases NIK and IKKα both act upstream of NF-κB2 and RelB and are involved in activation of the non-canonical NF-κB signaling^26^. Mice bearing a T cell specific deletion of IKKα show compromised Treg development^28^ whereas NIK deficiency leads to reduced Treg numbers in the periphery^29^. Mice lacking RelB display inflammatory cell infiltration in several organs, myeloid hyperplasia and splenomegaly due to extramedullary hematopoiesis. While these effects were seen to depend on T cells, the inflammation seen in *Relb*−/− mice was shown to be due to a role of RelB in non-hematopoietic cells^30^.

Signals activating the non-canonical NF-κB pathway induce processing of the NF-κB precursor protein p100, encoded by *Nfkb2*, into the mature NF-κB2 subunit p52. In resting cells, p100 sequesters RelB in the cytoplasm and signal-induced p100 processing allows for nuclear activation of RelB:p52 heterodimers^26^. However, p100 retains RelA- and cRel-containing heterodimers as well^31^. Indeed, p100 deficiency triggers sustained RelA activity in TLR4-stimulated embryonic fibroblasts^32^, and also prolongs cRel responses in maturing B cells^33^.

Interestingly, a lack of p100 in myeloma cells, attributed to genetic aberrations, provokes an alternate RelB:p50 NF-κB response via the canonical pathway^34^. RelB is encoded by an NF-κB target gene, and its expression is upregulated by canonical NF-κB signals^35^. In the absence of p100, de novo synthesized RelB accumulates in the nucleus as RelB:p50 heterodimers and regulates NF-κB-driven gene expression^34^. These studies underline a complex role of *Nfkb2* at the crossroad of canonical and noncanonical NF-κB pathways; p52 constitutes the noncanonical RelB:p52 activity while p100 inhibits the activity of multiple p50-containing NF-κB dimers, including RelB:p50 dimer, in the canonical pathway. Despite the involvement of *Nfkb2* in diverse physiological and pathological settings, the role of *Nfkb2*-dependent pathways in Treg biology has been less explored.

Here, we report that mice deficient in *Nfkb2* showed normal thymic development of Tregs, but enhanced frequencies of peripheral eTregs. This effect was cell-autonomous, since it was recapitulated in bi-parental chimeras of wild-type (WT) and *Nfkb2*−/− mice. These *Nfkb2*−/− Tregs exhibited normal suppressive function, showed greater survival, activation and proliferation *in vivo*, and trafficked better to non-lymphoid tissues. However, the frequencies of Tregs and eTregs were normal in bi-parental chimeras of WT and knockout mice lacking RelB, which regulates gene-expressions in association with p52. Our biochemical analyses revealed that *Nfkb2* deficiency in Tregs did not alter RelA and cRel activities but triggered instead an additional strong NF-κB activity comprising of RelB:p50 heterodimers. Together, our data indicate, as shown recently as well, that *Nfkb2* functions in Treg are dictated primarily by the inhibitory p100, which restrains RelB:p50 mediated Treg activation, and not by p52. Nuclear induction of RelB-p50 in the absence of p100 contributes to a greater prominence of activated Tregs. Our data provide further insights into the complex roles that p100 plays in Treg biology.

## Results

### Comparable thymic Treg development but higher splenic Treg numbers in Nfkb2−/− mice

**Figure 1:**
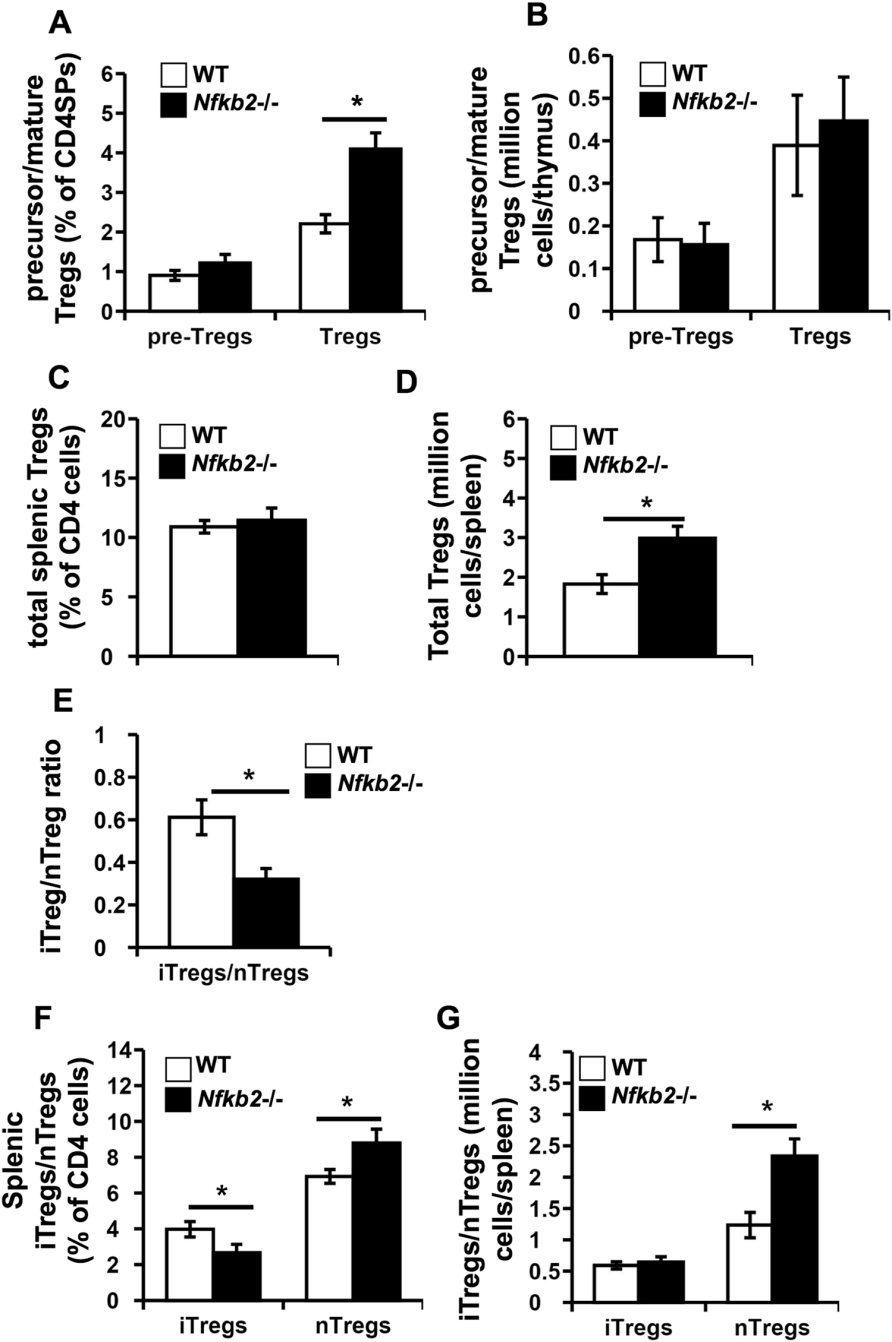
Unaltered thymic Treg numbers but higher splenic Treg numbers in Nfkb2−/− mice. (**A**,**B**) Thymocytes isolated from WT (B6.SJL) and *Nfkb2*−/− mice were fixed, permeabilised and stained with anti-mouse CD45.1, CD45.2, CD90, CD4, CD8, CD25 and FOXP3 to identify Treg precursors (pre-Tregs) and mature Tregs (Supplementary Fig. S1). The bar graphs represent the (**A**) frequencies and (**B**) cell numbers per thymus of pre-Tregs and mature Tregs in CD90^+^CD4^+^CD8^−^ thymocytes (CD4SP: CD4 single-positive thymocytes). (**C**–**G**) Spleen cells isolated from WT and *Nfkb2*−/− mice were fixed, permeabilised and stained with anti-mouse CD45.1, CD45.2, CD4, CD25, FOXP3 and HELIOS to identify Tregs as well as the iTreg and nTreg subpopulations (Supplementary Fig. S2). The bar graphs represent (**C**) the frequencies and (**D**) numbers per spleen of the Tregs in total CD4 cells. The ratio of iTreg/nTreg within the total Tregs (**E**), and the frequencies (**F**) and numbers per spleen (**G**) of iTregs and nTregs in the spleens of WT and *Nfkb2*−/− mice are also shown. *p < 0.05; n ≥ 12 mice/group.

Thymic Treg development has been shown to consist of two steps: a TCR- and co-stimulation-dependent step that generates thymic Treg precursors and a cytokine-dependent second step that results in the differentiation of Treg precursors into mature Tregs^36^. Thymic Treg precursors in WT (B6.SJL: expressing CD45.1) and *Nfkb2*−/− (expressing CD45.2) mice were identified as CD90^+^CD4^+^CD8^−^ (CD4 single positive: CD4SP) thymocytes expressing CD25 but not FOXP3, while mature thymic Tregs were identified as CD25^+^FOXP3^+^CD4SP thymocytes (Supplementary Information, Fig. S1)^36^. While thymi from WT and *Nfkb2*−/− mice had equivalent frequencies of Treg precursors, the frequency of Tregs was significantly higher in *Nfkb2*−/− mice than in WT mice (Fig. 1A). The cell numbers of Treg precursors and Tregs in the thymus of WT and *Nfkb2*−/− mice respectively were equivalent (Fig. 1B) Equivalent Treg numbers despite higher frequency of Tregs in *Nfkb2*−/− mice might partly be explained by a reduction in total thymic cell numbers seen in *Nfkb2*−/− mice.

In the periphery, the frequency of splenic Tregs (CD4^+^CD25^+^FOXP3^+^; Supplementary Information, Fig. S2)^37^ was comparable between WT and *Nfkb2*−/− mice but the latter contained significantly higher Treg numbers (Fig. 1C,D). This is likely to be a consequence of the higher numbers of CD4 T cells in *Nfkb2*−/− mouse spleens (Supplementary Information, Fig. S2). In addition to nTregs of thymic origin, the splenic Treg population also includes peripherally induced Tregs (iTregs) generated from conventional CD4 T cells. The transcription factor HELIOS appears to be generally expressed in thymic Tregs but not in iTregs^6^, although this is subject to caveats^38^. When we analyzed splenic Tregs as either HELIOS-high nTregs or HELIOS-low iTregs (Supplementary Information, Fig. S2), the ratio of iTregs to nTregs among the splenic Tregs in *Nfkb2*−/− mice was less than in WT mice (Fig. 1E). This was reflected in the reduced frequencies (Fig. 1F) of iTregs in the spleens of *Nfkb2*−/− mice compared to WT mice. In contrast, the frequencies and numbers of nTregs were increased in the spleens of *Nfkb2*−/− mice (Fig. 1F,G).

### Splenic Tregs in Nfkb2−/− mice have a higher proportion of effector Tregs

**Figure 2:**
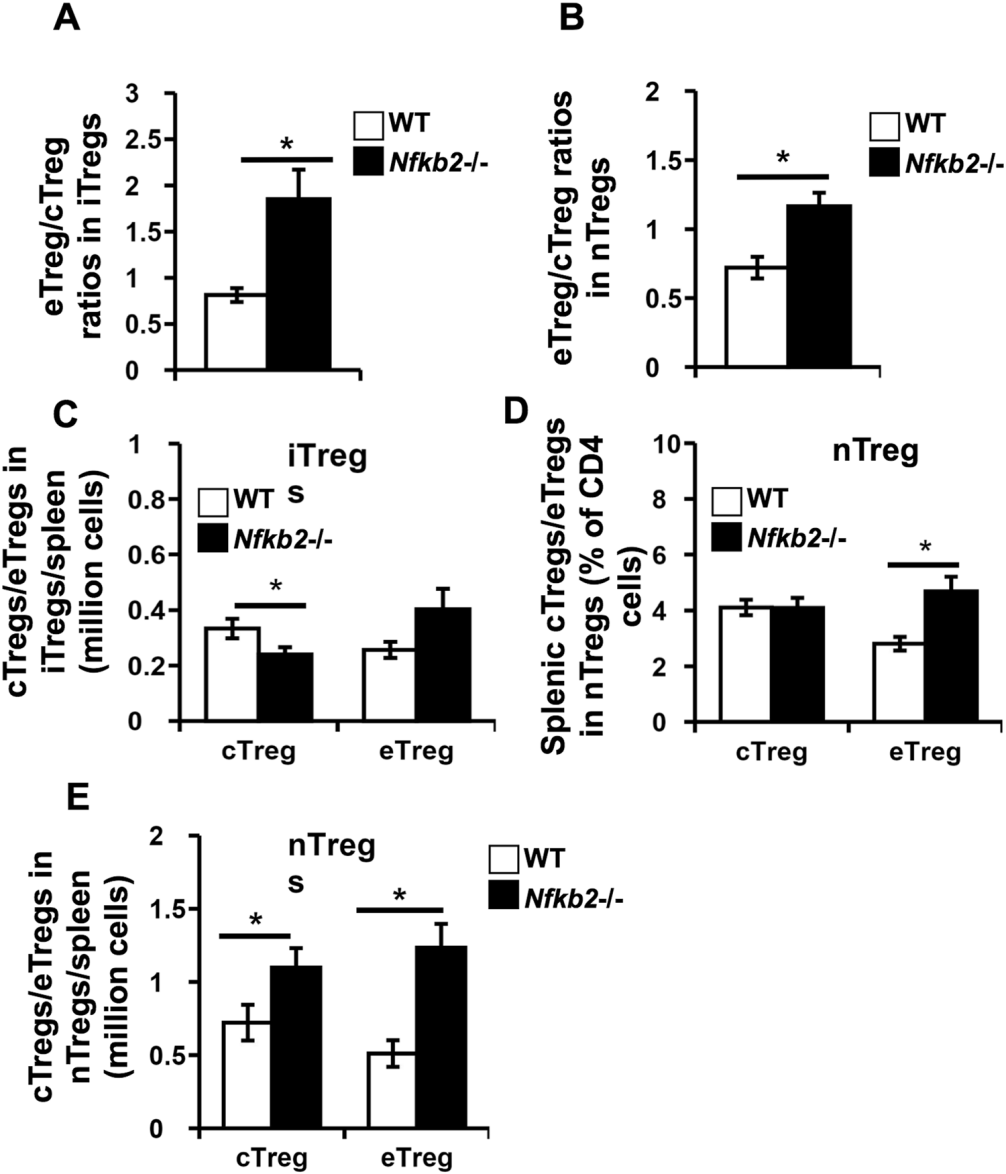
Nfkb2−/− mice have higher proportions of peripheral effector Tregs compared to WT mice. Spleen cells were isolated from WT and *Nfkb2*−/− mice, fixed, permeabilised and stained with anti-mouse CD4, CD25, FOXP3, HELIOS, CD62L and CD44 to identify Tregs and Treg subsets (Supplementary Fig. S2). (**A**,**B**) The bar graphs represent the ratios of effector (eTreg) to central (cTregs) phenotype Tregs among (**A**) iTregs and (**B**) nTregs. (**C**–**E**) The bar graphs represent the numbers per spleen of central and effector iTregs (**C**), and the frequencies (**D**) and numbers per spleen (**E**) of central and effector nTregs in WT and *Nfkb2*−/− mice. *p < 0.05; n = 15 mice/group.

In addition to developmental heterogeneity, peripheral Tregs can also be distinguished into central Tregs and effector Tregs, based on the expression of CD62L and CD44 (Supplementary Information, Fig. S2)^14^. CD62L^low^CD44^high^ effector Tregs were relatively more prominent than CD62L^high^CD44^low^ central Tregs in both iTreg (Fig. 2A) and nTreg (Fig. 2B) compartments in *Nfkb2*−/− spleens compared to their WT counterparts. Thus, it was notable that, while total splenic iTreg numbers were comparable in *Nfkb2*−/− mice and WT mice (Fig. 1G), the numbers of effector iTregs tended to be higher in *Nfkb2*−/− mice (Fig. 2C). As expected, both the frequencies (Fig. 2D) and numbers of effector nTregs (Fig. 2E) were higher in the spleens of *Nfkb2*−/− mice compared to WT mice.

### NF-κB2 has a cell intrinsic role in regulating the splenic Treg population

The data above regarding splenic Tregs in WT and *Nfkb2*−/− mice suggested a possible role of NF-κB2 in regulating both splenic iTreg population and the proportions of effector Tregs among both iTregs and nTregs. Defects in both thymic and splenic organization have been reported in *Nfkb2*−/− mice due to a critical role of the non-canonical NF-κB pathway in the stromal cell compartments involved^39,40^. Therefore, it was necessary to examine whether the observed effects of the *Nfkb2*−/− genotype on the Treg compartment were cell-intrinsic or not. For this, we generated bi-parental bone marrow chimeras using bone marrow cells from WT and *Nfkb2*−/− mice, and the Treg populations in the thymus and spleen of bi-parental bone marrow chimeras were analyzed after reconstitution.

**Figure 3:**
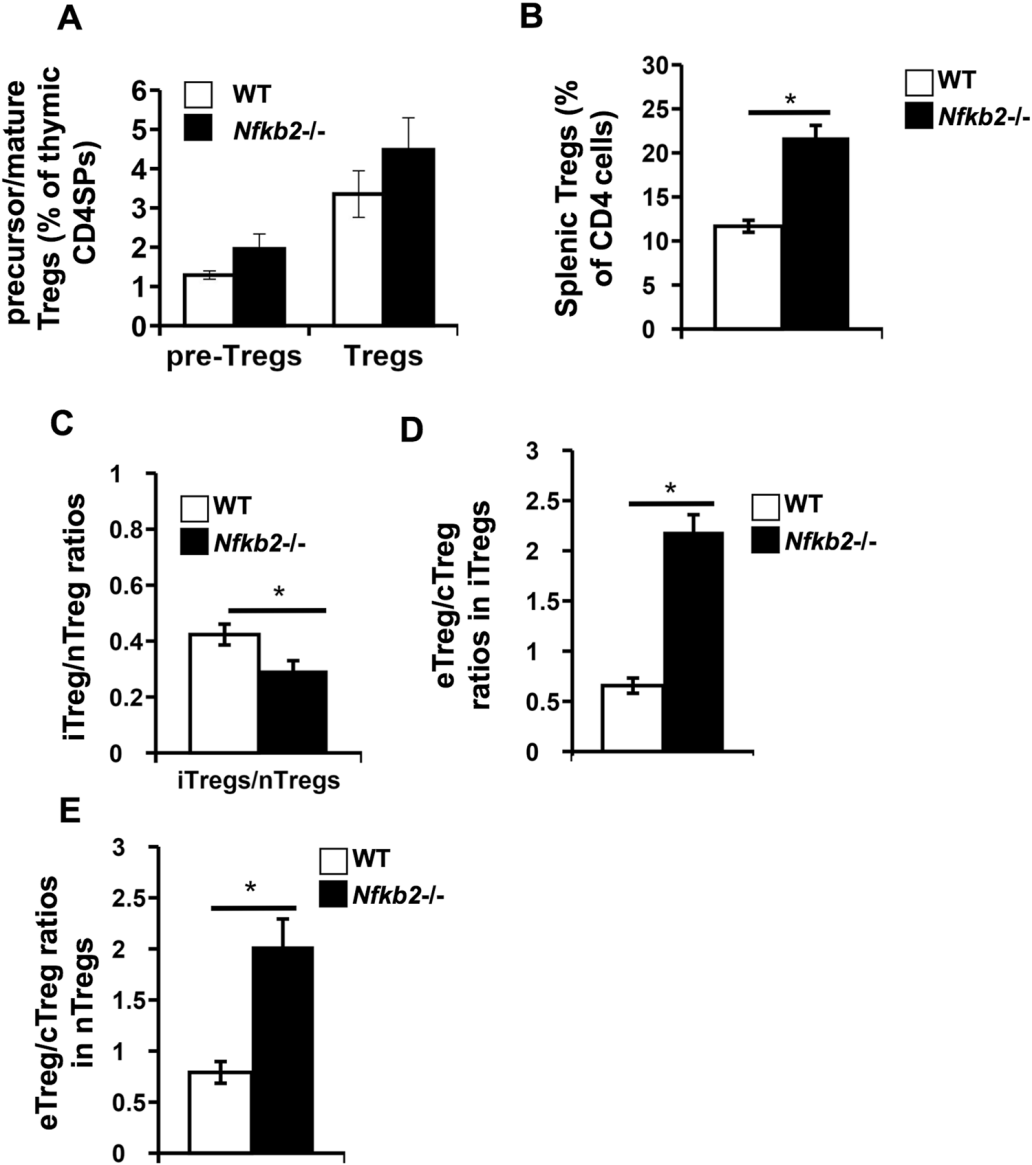
In bi-parental mixed bone marrow chimeras, the Nfkb2−/− genotype shows greater prominence of peripheral Tregs and higher proportions of effector Tregs. (**A**) Thymocytes were isolated from WT and *Nfkb2*−/− bi-parental bone marrow chimeras, fixed, permeabilized and stained to identify precursor (pre-Tregs) and mature Tregs (Supplementary Fig. S1). The bar graph shows the frequencies of pre-Tregs and mature Tregs in CD4SP thymocytes. (**B**–**D**) Spleen cells were isolated from WT and *Nfkb2*−/− bi-parental bone marrow chimeras and stained to identify Tregs and their subsets (Supplementary Fig. S2). The bar graphs show frequencies of total splenic Tregs (**B**), ratios of iTregs to nTregs (**C**), and the ratios of effector (eTregs) to central (cTreg) Tregs among iTregs (**D**) and nTregs (**E**) in the WT and *Nfkb2*−/− donors in bi-parental bone marrow chimeras. *p < 0.05; n ≥ 10 mice/group.

In the thymi of the bi-parental bone marrow chimeras, the frequencies of Treg precursors (pre-Tregs) and mature Treg were equivalent (Fig. 3A) between WT and *Nfkb2*−/− donors. In the spleen, there was a large increase in *Nfkb2*−/− Treg frequency compared to the WT Tregs (Fig. 3B). As in the parent strain, the ratio of iTregs to nTregs in splenic Tregs was lower in *Nfkb2*−/− Tregs (Fig. 3C). The effector and central Treg populations in the spleens of bi-parental bone marrow chimeras showed patterns similar to those observed in the parental strains, namely, an increase in the effector to central Treg ratio among both iTregs (Fig. 3D) and nTregs (Fig. 3E) in the *Nfkb2*−/− donor compared to WT donor. Thus, the absence of NF-κB2 led to reduced proportion of iTregs and higher effector Tregs in a cell-intrinsic manner.

### Differential expression of several characteristic Treg markers between WT and Nfkb2−/− Tregs

**Figure 4:**
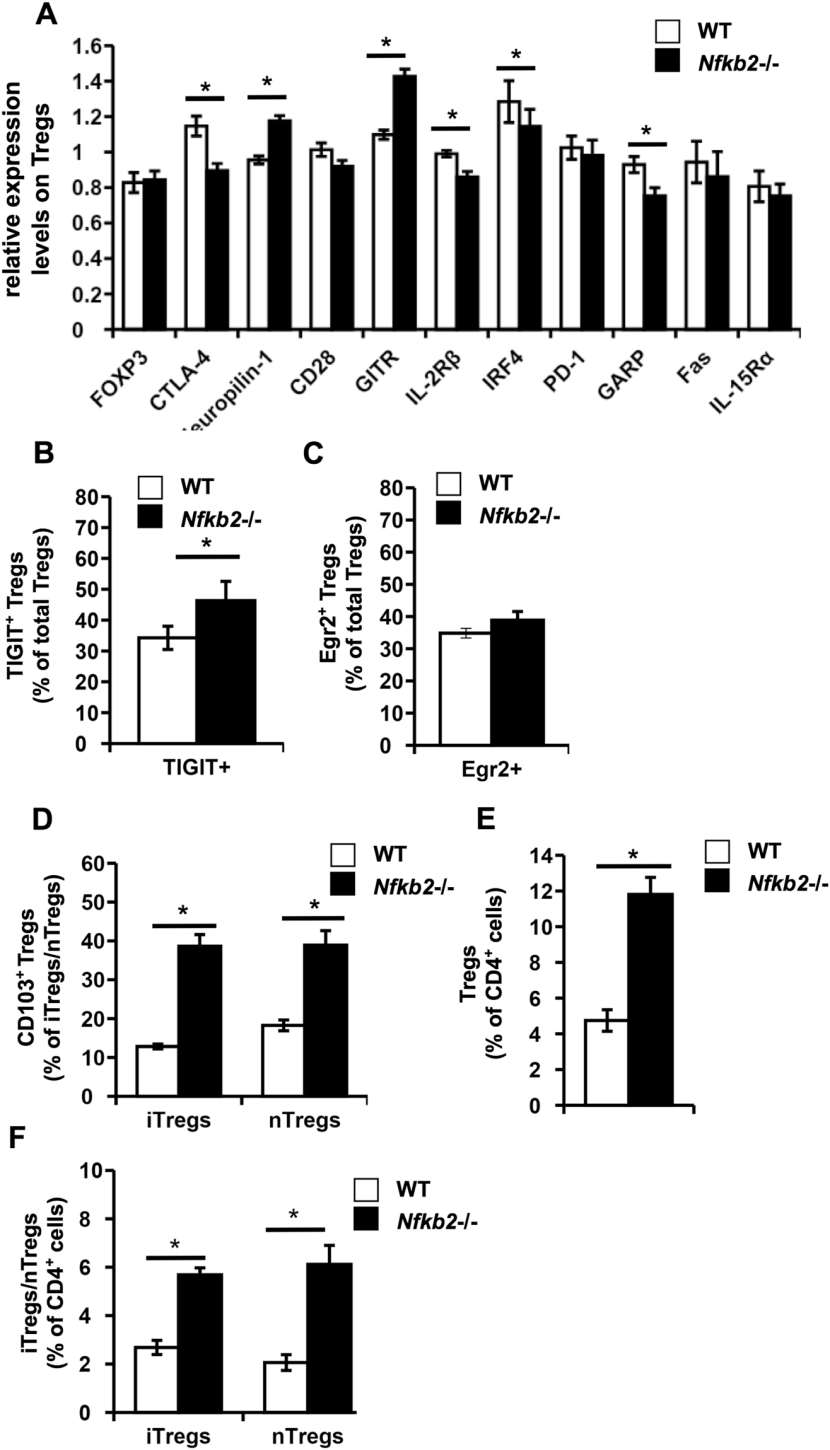
Qualitative differences between WT and Nfkb2−/− Tregs. (**A**–**D**) Spleen cells were isolated from the bi-parental bone marrow chimeras, fixed, permeabilised and stained with anti-mouse CD45.1, CD45.2, CD4, CD25, FOXP3 and either CD152 (CTLA-4), CD304 (Neuropilin-1), CD28, CD357 (GITR), CD122 (IL-2Rβ), IRF4, CD279 (PD-1), GARP, Fas(CD95), CD215 (IL-15Rα), TIGIT, Egr2 and CD103 (Supplementary Fig. S3). Mean fluorescence indices (MFI) of the indicated molecules were calculated for WT and *Nfkb2*−/− Tregs and normalized to WT. The bar graphs indicate the relative levels (MFI) of the indicated surface/intracellular markers expressed by WT versus *Nfkb2*−/− Tregs (**A**), frequencies of TIGIT^+^ total Tregs (**B**), frequencies of Egr2^+^ total Tregs (**C**), and frequencies of CD103^+^ iTregs and nTregs (**D**), among WT and *Nfkb2*−/− donors respectively in the bi-parental bone marrow chimeras. (**E**,**F**) Leukocytes were isolated from the liver of the bi-parental bone marrow chimeras, fixed, permeabilised and stained with anti-mouse CD45.1, CD45.2, CD4, CD25, FOXP3 and HELIOS to identify Tregs and their subsets (Supplementary Fig. S2). The bar graphs depict frequencies of total WT and *Nfkb2*−/− Tregs (**E**), as well as frequencies of both iTregs and nTregs (**F**) in the liver of the bi-parental bone marrow chimeras. *p < 0.05; n ≥ 5 mice/group.

Since NF-κB2 regulated the relative prominence of specific Treg sub-populations, we tested if the absence of NF-κB2 also led to alterations in the phenotype of these Tregs. We therefore examined the expression of Treg specific transcription factor FOXP3^1^, molecules contributing to Treg suppressive function namely CTLA-4 (CD152)^41^, Neuropilin-1 (CD304)^42,43^ and CD28^44^, markers highly expressed by effector Tregs or representative of Treg activation such as GITR (CD357), IL-2Rβ (CD122)^11^, IRF4, TIGIT, Egr2, PD-1 (CD279)^20^, GARP^45^, Fas (CD95) and IL-15Rα (CD215) which plays a role in Treg proliferation^46^ on Tregs from the bi-parental bone marrow chimeras (Supplementary Information, Fig. S3). Of the markers analyzed, the expression of FOXP3, CD28, PD-1, Fas and IL-15Rα was found to be unaltered between WT and *Nfkb2*−/− Tregs (Fig. 4A). The expression of Neuropilin-1 and GITR was found to be higher whereas the expression of CTLA-4, IRF4, IL-2Rβ and GARP was lower on *Nfkb2*−/− Tregs compared to WT Tregs (Supplementary Information; Fig. S3 and Fig. 4A). The expression of TIGIT and Egr2 on Tregs displayed a bimodal distribution, as reported previously^20^, and hence the frequencies of TIGIT^+^ and Egr2^+^ WT and *Nfkb2*−/− Tregs respectively in the bi-parental bone marrow chimeras were analyzed (Supplementary Information, Fig. S3). While the frequencies of TIGIT^+^ cells were found to be higher among *Nfkb2*−/− Tregs, the frequency of Egr2^+^ cells were comparable between WT and *Nfkb2*−/− Tregs (Fig. 4B,C).

Additionally, CD103^+^ Tregs constitute a subset of eTregs^47^ and as the overall eTreg compartment was expanded among *Nfkb2*−/− Tregs, we further analyzed the CD103^+^ Treg compartment (Supplementary Information, Fig. S3). Interestingly, we observed that both *Nfkb2*−/− iTregs and nTregs showed higher proportions of CD103^+^ cells compared to the corresponding WT populations (Fig. 4D). Effector Tregs are reported to make up the majority of Tregs present in non-lymphoid organs^12^. As the proportion of effector Tregs among the *Nfkb2*−/− Tregs was higher compared to WT Tregs in a cell-intrinsic manner, we hypothesized that there would be a higher representation of these *Nfkb2*−/− Tregs in non-lymphoid organs such as liver in the bi-parental bone marrow chimeras. Indeed, we found that there was a higher frequency of *Nfkb2*−/− Tregs than that of WT Tregs in the liver (Fig. 4E) of the mixed bone marrow chimeras, and this included both nTregs and iTregs (Fig. 4F).

### Generation and functions of Nfkb2−/− Tregs

**Figure 5:**
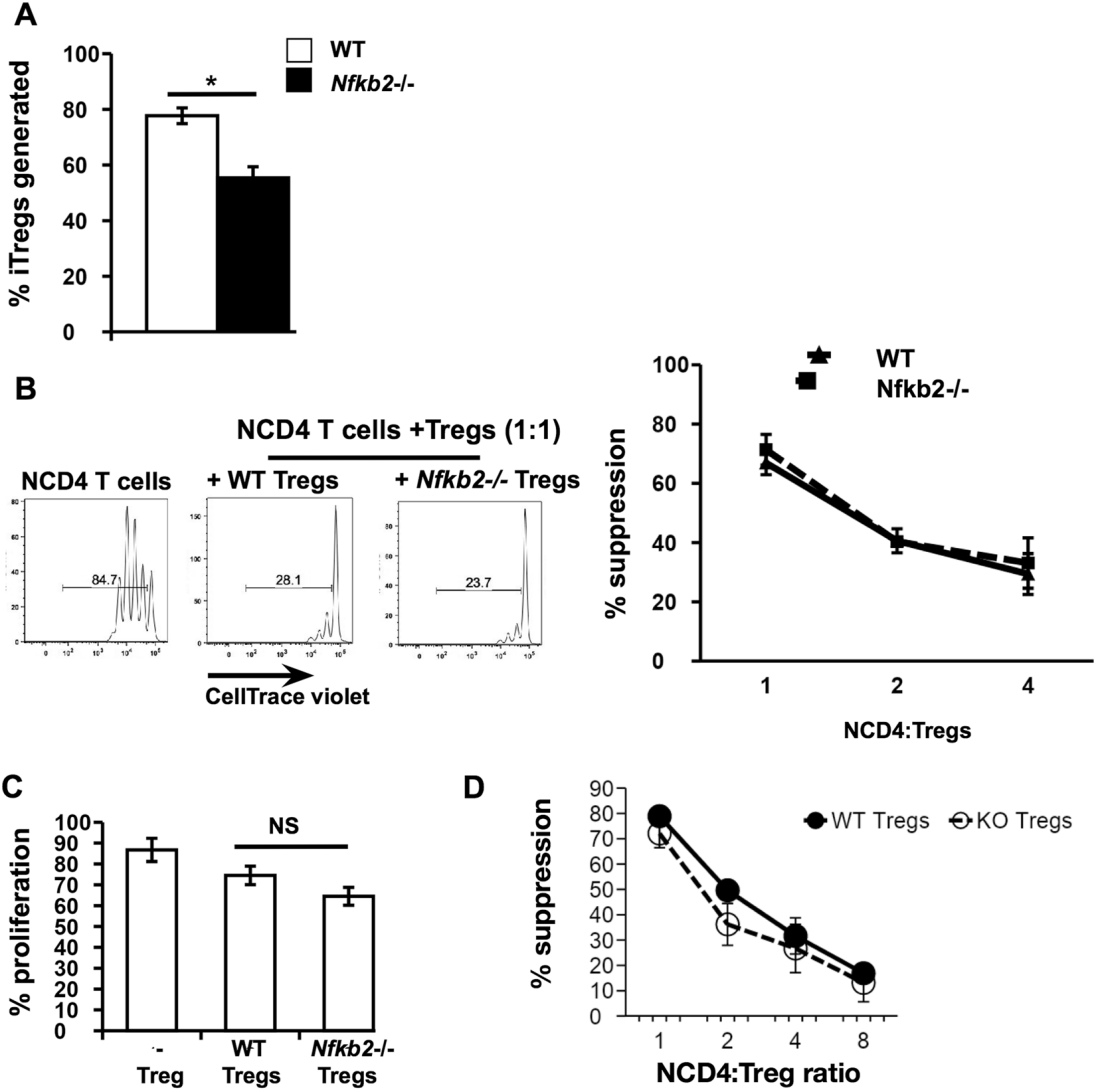
Nfkb2−/− CD4 T cells show poor Treg generation *in vitro*, but Nfkb2−/− Tregs show normal suppressive capacity. (**A**) WT and *Nfkb2*−/− naïve CD4 (NCD4) T cells were sorted from bi-parental bone marrow chimeras and stimulated *in vitro* under Treg differentiation conditions (Supplementary Information, Fig. S4). The bar graph indicates frequencies of Tregs generated *in vitro* from WT and *Nfkb2*−/− NCD4 T cells. The figure is an average of 3 independent experiments. (**B**) NCD4 T cells isolated from B6.GFP mice labelled with CellTrace violet dye were cultured with CD90^−^ C57BL/6 irradiated spleen cells and aqueous-phase anti-mouse CD3 (2 μg/ml) in the absence or presence of WT or *Nfkb2*−/− Tregs isolated from the spleens of bi-parental bone marrow chimeras at varying responder to suppressor ratios for 60 h. NCD4 T cell proliferation was then analysed by CellTrace violet dye dilution. The line graph represents the percentage of suppression at each responder to suppressor ratio calculated as [(%proliferation without Tregs − %proliferation with Tregs)/(%proliferation without Tregs) × 100] with % suppression mediated by WT Tregs represented as solid and *Nfkb2*−/− Tregs as dashed lines respectively. The figure is an average of 3 independent experiments. (**C**) NCD4 T cells were isolated from B6.GFP mice, labelled with CellTrace violet dye and transferred into TCRβδ−/− mice either alone or with WT or *Nfkb2*−/− Tregs respectively for 3 days. The proliferation of B6.GFP NCD4 T cells was assessed in the spleens of recipient mice 3 days after transfer to estimate the *in vivo* suppressive effects of WT and *Nfkb2*−/− Tregs. n = 3mice/group. (**D**) NCD4 T cells isolated from B6.GFP mice labelled with CellTrace violet dye were cultured with CD90^−^ C57BL/6 irradiated spleen cells and aqueous-phase anti-mouse CD3 (2 μg/ml) in the absence or presence of Tregs isolated from the spleens of WT or *Nfkb2*−/− mice at varying responder to suppressor ratios for 60 h. NCD4 T cell proliferation was then analysed by CellTrace violet dye dilution. The line graph represents the percentage of suppression at each responder to suppressor ratio calculated as [(%proliferation without Tregs − %proliferation with Tregs)/(%proliferation without Tregs) × 100] with % suppression mediated by WT Tregs represented as solid and *Nfkb2*−/− Tregs as dashed lines respectively. The figure is an average of 2 independent experiments.

The iTreg compartment was reduced in *Nfkb2*−/− Tregs compared to WT Tregs, both in the parental strain and in the bi-parental bone marrow chimeras. The reduction indicated a possible role of NF-κB2 in the generation and/or survival of iTregs. To test the former possibility, WT and *Nfkb2*−/− naïve CD4 (NCD4) T cells were sorted from bi-parental bone marrow chimeras and cultured *in vitro* under Treg differentiation conditions (Supplementary Information; Fig. S4). At the end of five days, it was observed that the *Nfkb2*−/− NCD4 T cells gave rise to a lower frequency of Tregs *in vitro* compared to WT NCD4 T cells (Fig. 5A). FOXP3 staining profiles were comparable between WT and *Nfkb2*−/− iTregs (Supplementary Information; Fig. S4).

Since the *Nfkb2*−/− Tregs contained a higher proportion of effector Tregs that have been shown to be potent producers of IL-10^21^, we tested the ability of WT and *Nfkb2*−/− Tregs to suppress NCD4 T cell proliferation. Interestingly, the *Nfkb2*−/− Tregs showed equivalent suppression of naïve T cell proliferation *in vitro* compared to WT Tregs (Fig. 5B). In addition, when we compared the ability of bi-parental bone marrow chimera-derived WT and *Nfkb2*−/− Tregs to suppress NCD4 T cell proliferation upon transfer in TCRβδ−/− mice, for a duration of 3 days, the *in vivo* suppressive function of WT and *Nfkb2*−/− Tregs was also found to be comparable (Fig. 5C). Additionally, parent strain-derived WT and *Nfkb2*−/− Tregs also showed equivalent capacity to suppress NCD4 T cell proliferation *in vitro* (Fig. 5D).

### The Nfkb2−/− Tregs show altered survival, proliferation and effector differentiation

**Figure 6:**
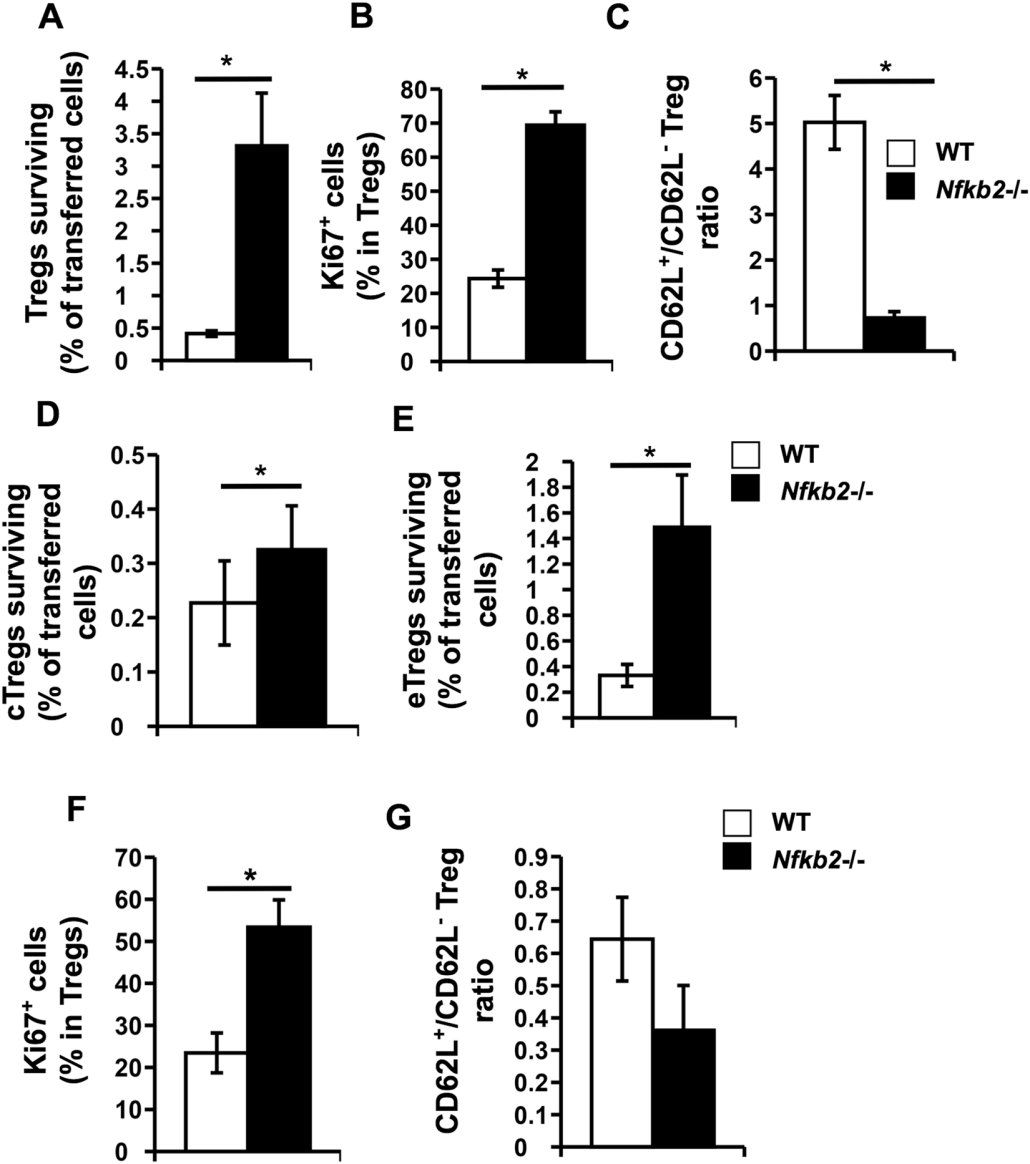
Nfkb2−/− central and effector Tregs show higher survival, activation and proliferation *in vivo*. (**A**–**C**) WT and *Nfkb2*−/− central Tregs were sorted from the spleens of bi-parental bone marrow chimeras and parked *in vivo* for 10 days. Transferred populations were then analysed (Supplementary Fig. S5). Bar graphs represent (**A**) frequencies of the indicated transferred Treg populations in the spleen of recipients at the end of 10 days, (**B**) frequencies of proliferating cells in respective transferred Treg population as indicated by Ki67 expression frequencies, and (**C**) ratios of central (CD62L^high^) to effector (CD62L^low^) Tregs. (**D**) WT and *Nfkb2*−/− central Tregs were sorted from the spleens of bi-parental bone marrow chimeras and parked *in vivo* for 3 days. The bar graph represents the frequencies of the indicated transferred Treg populations in the spleen of recipients at the end of 3 days. (**E**–**G**) WT and *Nfkb2*−/− effector Tregs were sorted from the spleens of bi-parental bone marrow chimeras and parked *in vivo* for 10 days as above. Bar graphs represent (**E**) frequencies of the indicated transferred Treg populations in the spleens of recipients at the end of 10 days, (**F**) frequencies of proliferating cells in respective transferred Treg populations as indicated by Ki67 expression frequencies, and (**G**) ratios of central (CD62L^high^) to effector (CD62L^low^) Tregs. *p < 0.05; n ≥ 4 mice/group.

The higher proportions of *Nfkb2*−/− donor Tregs in the bi-parental chimeras could be plausibly due to differential survival and/or proliferation and activation of *Nfkb2*−/− central and effector Tregs compared to WT Tregs. We first compared survival, proliferation and activation of WT (CD45.1^+^) and *Nfkb2*−/− (CD45.2^+^) central Tregs (CD4^+^CD25^+^CD62L^high^) sorted from bi-parental bone marrow chimeras by transferring them into recipient congenic mice in equal numbers (Supplementary Information; Fig. S5). Higher frequencies of *Nfkb2*−/− Tregs was observed in the spleen 10 days post-transfer (Fig. 6A) Proliferation among transferred Tregs at ten days post-transfer was also estimated using Ki67 detection, which showed that a higher proportion of transferred *Nfkb2*−/− Tregs were Ki67^+^ compared to WT Tregs (Fig. 6B). In addition to displaying greater proliferation, it was evident that *Nfkb2*−/− cTregs showed greater conversion to an effector phenotype (Fig. 6C).

In order to distinguish survival versus proliferation differences between WT and *Nfkb2*−/− cTregs we assessed the survival of WT and *Nfkb2*−/− cTregs *in vivo* 3 days after transfer, a time point at which there is neither major detectable proliferation nor transition to an effector phenotype (Supplementary Information, Fig. S6)^11^, and observed that *Nfkb2*−/− cTregs displayed enhanced survival compared to WT Tregs (Fig. 6D). Nonetheless, these data do not account for differences potentially contributed by differences in proliferation below detection limits for this assay.

Thus, central-phenotype *Nfkb2*−/− Tregs showed better survival, proliferation and effector transition compared to WT central Tregs. We next tested if effector-phenotype Tregs also showed similar differences, and if re-conversion from the effector to central phenotype differed between the two genotypes. To examine these issues, WT (CD45.1^+^) and *Nfkb2*−/− (CD45.2^+^) effector Tregs (CD4^+^CD25^+^CD62L^low^) purified from bi-parental bone marrow chimeras were parked *in vivo* as above (Supplementary Information; Fig. S5). Ten days later, the transferred populations were analyzed in the spleens of recipient mice. Similar to the observation in central Tregs, *Nfkb2*−/− effector Tregs showed enhanced survival and proliferation (Fig. 6E,F). Interestingly, when the effector/central phenotype was analyzed in the transferred effector Tregs, it was evident that higher frequencies of WT effector Tregs shifted to a central phenotype, despite proliferating and/or surviving less well (Fig. 6G). Thus, NF-κB2 in Tregs appears to control the efficiency of survival, proliferation and transition between resting and effector phenotypes.

### Altered NF-κB activity in Nfkb2−/− Tregs

**Figure 7:**
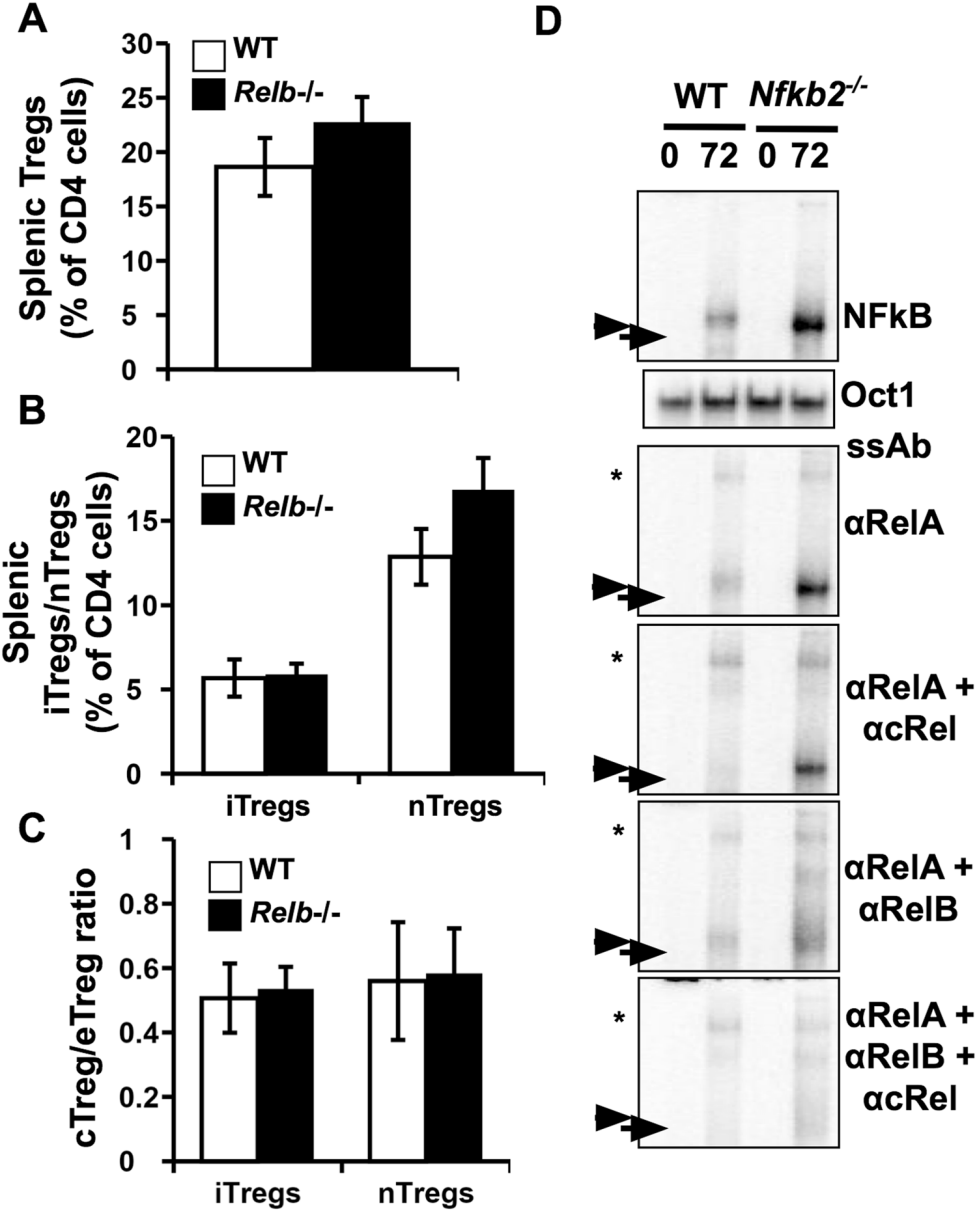
RelB activity is likely responsible for altered Treg homeostasis in the absence of Nfkb2. (**A**–**C**) Tregs and Treg subsets in the spleens of bi-parental bone marrow chimeras of WT and *Relb*−/− mice were identified (Supplementary Fig. S2) and their frequencies estimated. The bar graphs represent frequencies of (**A**) total Tregs, (**B**) iTregs and nTregs, and (**C**) ratios of central to effector Tregs among the iTregs and nTregs present in the spleen of the bi-parental bone marrow chimeras of WT and *Relb*−/− mice. n = 7 mice/group. (**D**) Tregs were generated *in vitro* from WT and *Nfkb2*−/− NCD4 T cells under Treg-inducing conditions using anti-CD3 + anti-CD28 + TGFβ + IL-2 for 72 h as described, and subjected to an EMSA to assess nuclear NF-κB activity. Supershift assays were done with anti-RelA, anti-RelA + anti-cRel and anti-RelA + anti-RelB antibodies to identify the relative prominence of RelA, RelB and cRel in these assays. Arrow and arrowhead represent RelA-, RelB- and cRel- containing NF-κB activity, respectively. Asterisk represents supershifted complexes. The panels for detection of NF-κB activity and the relative prominence of RelA, RelB and cRel by means of supershifts are a part of a single gel (shown in Supplementary Fig. S7). The panel for loading control, using Oct1, is from a separate gel (also depicted in Supplementary Fig. S7). ssAb:supershift antibody. The data represent two independent experiments.

The observations that *Nfkb2*−/− Tregs showed enhanced survival, proliferation and activation compared to WT Tregs indicated a negative regulatory role for NF-κB2 in these contexts. *Nfkb2* deficiency causes loss of both p52 and p100. Because p52 predominantly functions as RelB:p52 heterodimers during non-canonical signaling^26^, we first asked if a lack of RelB similarly affected the frequencies of total Tregs and specific Treg subsets in the spleens of bi-parental chimeras of WT and *Relb*−/− parental strains. We found that the frequency of splenic *Relb*−/− and WT Tregs were not significantly different (Fig. 7A). Additionally, the frequencies of iTreg and nTreg populations were also comparable (Fig. 7B). Further, the distribution of cTregs and eTregs was comparable between WT and *Relb*−/− genotypes in both the iTreg and nTreg populations (Fig. 7C). These results indicated that *Nfkb2* functions in Tregs were independent of RelB:p52 and that chronic non-canonical signaling only marginally contributed in the observed eTreg phenotype.

We then investigated if *Nfkb2* deficiency triggered anomalous activation of p50-containing NF-κB heterodimers because of a lack of p100-mediated inhibition.

To this end, we examined nuclear extracts derived from WT and *Nfkb2*−/− Tregs in the electrophoretic mobility shift assay (EMSA) for NF-κB DNA binding activities and distinguished between RelA-, cRel- and RelB-containing heterodimers using antibody-mediated supershift assays (Supplementary Information, Fig. S7; Fig. 7D). While NF-κB activity was detectable in both WT and *Nfkb2*−/− Tregs, the latter showed strikingly higher nuclear NF-κB activity. Supershift assays showed that NF-κB activity was mainly composed of RelA- and cRel-containing dimers in WT Tregs. In the *Nfkb2*−/− Tregs, however, the high NF-κB activity was mainly due to RelB-containing dimers with a minimal contribution of cRel-containing dimers. Our data thus indicate that increased nuclear RelB:p50 activity in *Nfkb2*−/−Tregs, in the absence of p100, may be responsible for their higher activation, proliferation and survival. In other words, p100-mediated inhibition of RelB activation, rather than p100 processing-dependent activation of RelB:p52, determines *Nfkb2* functions in Tregs.

### Effects of Nfkb2 deficiency on the Treg transcriptome

**Figure 8:**
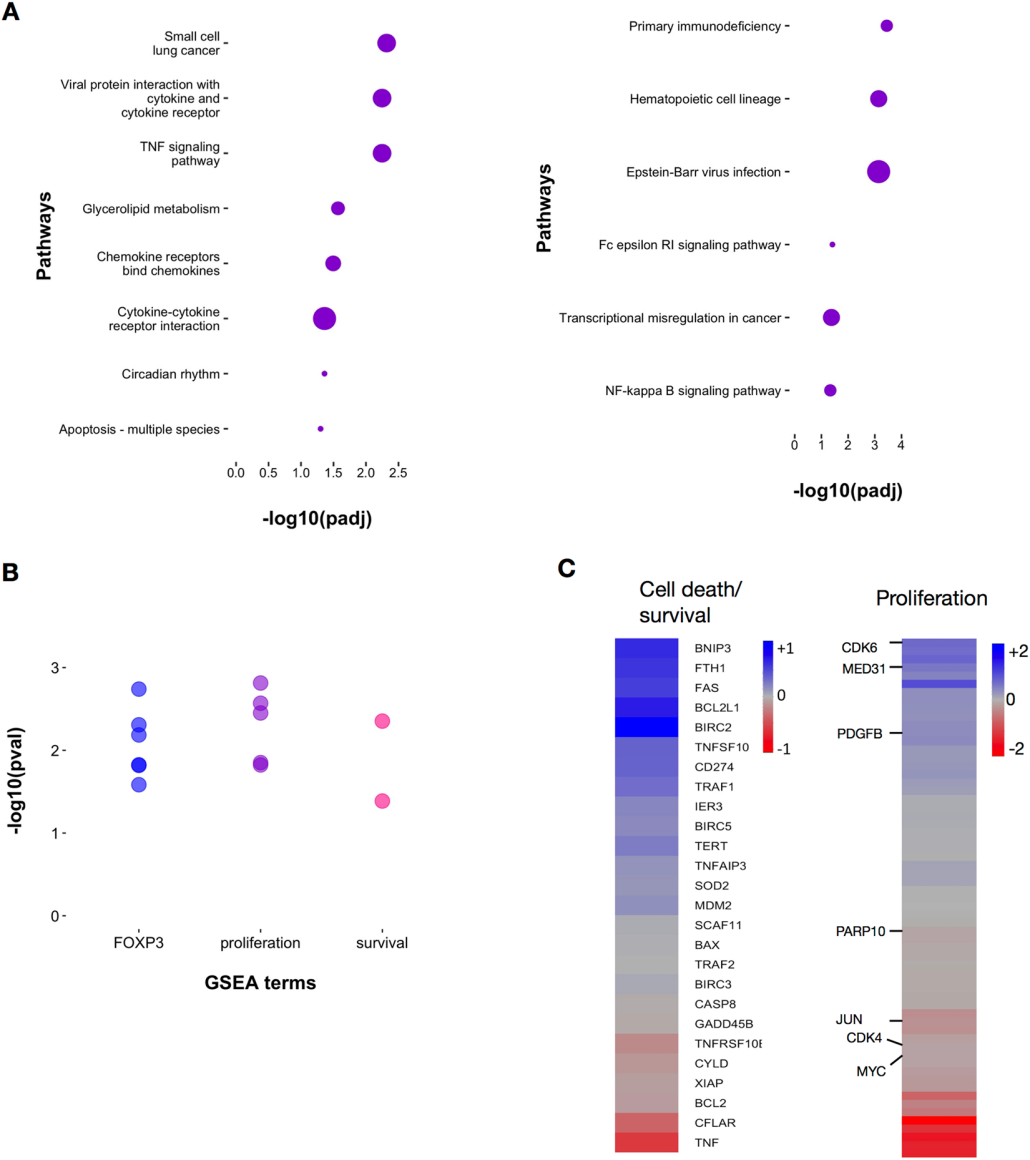
Transcriptome analysis shows misregulation of multiple pathways in Nfkb2−/− Tregs. (**A**) KEGG pathways enriched in *Nfkb2*−/− Tregs, - upregulated genes (left panel) and downregulated genes (right panel), - are plotted. Pathway analysis was done using g:profiler. (**B**) Gene sets related to cell proliferation, cell survival/death, and Foxp3 targets that are significantly enriched in *Nfkb2*−/− cells are plotted against their -log10(p-value). (**C**) Heatmap showing log2-fold changes of genes associated with cell survival/death and cell proliferation in *Nfkb2*−/− Tregs compared to WT cells. Genes associated with cell survival and proliferation are annotated. Genes were selected from the gene sets enriched in GSEA analysis.

We also evaluated the differences in gene expression between WT and *Nfkb2*−/− Tregs by RNA-seq analysis. It must be noted that these analyses were done *ex vivo*, with no further activation of the isolated Tregs *in vitro* as previously reported^48^, and therefore the outcomes of the two transcriptome analyses are complementary rather than simply confirmatory. Some 854 genes exhibited differential expression between WT and *Nfkb2*−/− Tregs (Supplementary Information, Table S1). We further investigated the enrichment of pathways annotated in KEGG database^49^ as well as Reactome database^50^ among these differentially expressed genes (Fig. 8A). Chemokine and cytokine signaling pathways, the TNF signaling pathway and apoptosis-related pathways were enriched in the set of genes upregulated in *Nfkb2*−/− Tregs, while primary immunodeficiency related pathways, hematopoietic cell-lineage pathways, and the NF-κB pathway were enriched in the downregulated gene set. Since *Nfkb2*−/− Tregs showed better survival, we asked if any of the curated gene sets in MolSigDB that are associated with cell proliferation or survival were enriched among differentially expressed genes. When we examined the enrichment of curated gene sets in MSigDB using GSEA software, we noted that four gene sets associated with cell proliferation and two terms related to cell survival/cell death were substantially enriched in *Nfkb2*−/− Tregs (Fig. 8B). Interestingly, our analyses revealed that gene sets associated with FOXP3 functions were also enriched in genes differentially expressed between WT and *Nfkb2*−/− Tregs (Fig. 8B). We then analyzed the RNA-seq data for the expression levels of several known genes associated with cell survival and cell proliferation (Fig. 8C). A comparison showed that several pro-survival genes, including those encoding Traf1^51^ and cIAP1^52^ (Birc2) were upregulated in *Nfkb2*−/− Tregs (Fig. 8C). Conversely, some pro-apoptotic genes, such as Bim, Bid and Bad^53^, were downregulated in the absence of Nfkb2-p100 (Fig. 8C). However, *Nfkb2* deficiency did not substantially alter the expression of cIAP2 (Birc3)^52^, Bcl2 and Mcl1^53^. Similarly, WT and *Nfkb2*−/− Tregs expressed almost equivalent levels of some genes such as Bax and Bak1^53^ (Supplementary Information, Fig. S8). Finally, we noted that pro-proliferative genes, such as Cdk6^54^ and Med31^55^, were also upregulated in *Nfkb2*−/− Tregs (Fig. 8C). These observations suggested that complex interactions between different pathways involved in cell survival likely dictates the *Nfkb2*−/− Treg phenotype.

## Discussion

The NF-κB signaling pathways occupy an important place in Treg biology. The canonical NF-κB signaling axis promotes Treg development and is also important in maintaining the peripheral Treg population. Absence of IKKβ, cRel or RelA leads to a reduction in the thymic Treg numbers^56,57^, implicating the importance of canonical NF-κB axis in Treg development. Further, p50−/− mice show a reduction in splenic Treg numbers^57^, extending the significance of the canonical NF-κB pathway in regulating peripheral Tregs. Both cRel and RelA also regulate peripheral Treg homeostasis and function with some redundancy between them, and a deficiency of either cRel or RelA in Tregs leads to compromised Treg function as well as lymphoproliferative disease^58^.

Non-canonical NF-κB signaling also plays a role in Treg development and homeostasis. NIK- or IKKα-deficient mice show a reduction in thymic Treg numbers, although the effect of the NIK deficiency appears to be Treg-extrinsic^28,29^. Nonetheless, the absence of NIK does result in reduced peripheral Treg numbers^29^. T cell-specific deletion of IKK-alpha also results in reduced Treg proliferation and suppressive function *in vivo*^28^. However, the kinases NIK and IKKα can also activate canonical NF-κB signaling^59^; and hence, Treg alterations in the absence of these kinases might not reflect direct effects on non-canonical NF-κB signaling. We have therefore extended these studies by analyzing mice with a deletion of the *Nfkb2* gene, which plays a critical role in non-canonical NF-κB signaling. Our results both confirm and extend recently reported findings^48,60^.

The thymic development of Tregs was largely comparable between WT and *Nfkb2*−/− mice. However, the prominence of splenic iTregs was notably lower in the *Nfkb2*−/− mice. The difference persisted in bi-parental bone marrow chimeric mice, indicating that the effect was likely cell-autonomous. This suggested that iTreg induction from conventional NCD4 T cells would be less efficient in the absence of functional NF-κB2 in those cells. Our data from *in vitro* generation of iTregs validates this expectation. The crosstalk involving the NF-κB2 protein and TGF-β signaling involved in iTreg induction remains to be elucidated.

The peripheral Treg phenotype of *Nfkb2*−/− mice was also notable for higher proportions of splenic effector Tregs. Despite the reduction in iTreg prominence, this effector phenotype was observed in both nTregs and iTregs. Again, the difference remained quite evident in bi-parental bone marrow chimeric mice as well, indicating a cell-autonomous effect of NF-kB2 deficit. Moreover, higher *Nfkb2*−/− Treg frequencies were also found in the liver of bi-parental bone marrow chimeras, consistent with the greater prominence of *Nfkb2*−/− effector Tregs. In addition, the proportions of CD103-expressing Tregs, which constitute a subpopulation of the effector Tregs that have been reported to be more efficient at suppressing T cell-induced colitis^47^, were also found to be higher in the *Nfkb2*−/− splenic Tregs.

In addition to these quantitative differences, *Nfkb2*−/− Tregs also differed from WT Tregs in the levels of expression of Treg activation-associated molecules as well as of molecules highly expressed on effector Tregs. The levels of GITR on *Nfkb2*−/− Tregs and the frequencies of TIGIT^+^ *Nfkb2*−/− Tregs were strikingly higher than WT Tregs consistent with the higher proportions of effector Tregs seen in the former^20^. Nonetheless, expression of other surface molecules known to show higher expression on eTregs, such as IL-2Rβ^11^ and GARP^45^, were found to be reduced on *Nfkb2*−/− Tregs. The levels of IRF4 were also reduced in *Nfkb2*−/− Tregs albeit modestly, though IRF4 is known to be involved in the expansion of eTregs^21^. The higher Neuropilin-1 expression on *Nfkb2*−/− Tregs may also in part reflect changes in iTreg and nTreg distribution, since Neuropilin-1 has been proposed to be a marker of nTregs^61^. The expression of molecules contributing to Treg suppressive functions, namely CTLA-4^41^, CD28^44^ and Neuropilin-1^42,43^, also showed diverse patterns. CD28 levels were comparable between WT and *Nfkb2*−/− Tregs, while there was higher expression of Neuropilin-1 and lower CTLA-4 expression in *Nfkb2*−/− Tregs. Despite lower levels of CTLA-4, *Nfkb2*−/− Tregs showed unaltered capacities compared to WT Tregs to suppress NCD4 T cell proliferation *in vitro* and *in vivo*. These data suggest that the absence of NF-kB2, in addition to increasing eTreg frequencies, leads to qualitative, aberrant changes in *Nfkb2*−/− Tregs, with consequences for their activation as well as functional status.

Notably, a recent report showed impaired *Nfkb2*−/− Treg function in older mice, associated with immune inflammation^48^, while the present data showed no impairment. It is thus possible that *Nfkb2*−/− Tregs begin to be compromised in their suppressive functions over time, rather than being poorly functional ab initio, and such a role for *Nfkb2* in maintaining Treg responsiveness over time would be of interest.

Interestingly, *Nfkb2*−/− Tregs, both cTregs and eTregs, survived better and underwent greater proliferation *in vivo*. Further, *Nfkb2*−/− cTregs converted to eTregs more efficiently, while *Nfkb2*−/− eTregs displayed lower reversion to the cTreg phenotype *in vivo*. These data help explain the Treg phenotype in *Nfkb2*−/− mice, and indicate that *Nfkb2* likely contributes a net negative effect on Treg proliferation, activation and survival, although our data do not provide definitive evidence for separate effects on each of these parameters. All these factors possibly contribute to the expansion of Tregs in total and eTregs in particular seen in the bi-parental chimeras within the *Nfkb2*−/− donor.

Thus, while the absence of NIK and IKKα led to a reduction in Treg numbers in the spleen^28,29^, the absence of *Nfkb2*−/− actually led to Treg expansion. Notably, RelB deficiency did not recapitulate this phenotype. This indicated differential modes of action of NIK and IKKα in Tregs and suggested that they act by means of activating the canonical NF-κB pathway rather than through the activity of RelB:p52 dimers. Thus, these results are consistent at first glance with the mechanistic possibility that the non-canonical NF-κB pathway negatively regulates processes involved in promoting Treg activation, proliferation and survival.

In addition to the role of NF-κB2/p100 in mediating non-canonical NF-κB signaling, it is also known to be able to sequester the major mediators of both the canonical and the non-canonical NF-κB pathways, RelA, cRel and RelB, in the cytoplasm, via its C-terminal ankyrin repeats^26,31^. Thus, NF-κB2/p100 can also function as an inhibitor of both NF-κB pathways. There is evidence that NF-κB2/p100 plays an inhibitory role in TCR-mediated canonical NF-κB activation^62,63^. The generation of effector Tregs is dependent on signaling through the TCR, with the likely involvement of cRel-dependent canonical NF-κB signaling^19,20^. Additionally, both cRel^25^ and RelA^24^ have been shown to regulate homeostasis of effector Tregs. Thus, it is possible that the absence of NF-κB2/p100 in Tregs can result in enhanced canonical NF-κB activity via RelA and cRel. This could well explain the increased proliferation, activation and survival of effector Tregs.

When we tested this possibility by generating bi-parental chimeras of WT and *Relb*−/−mice, it was evident that Tregs of the *Relb*−/− genotype did not show any expansion of peripheral Tregs or any increase in the prominence of effector Tregs. Thus, non-canonical RelB-dependent NF-κB activity does not provide any negative regulatory signals to Tregs. It is thus plausible that NF-κB2/p100 primarily functions non-redundantly as a negative regulator of NF-κB activity. The NF-κB2/p100 protein is typically proteolytically processed to p52, which dimerizes with RelB to complete non-canonical NF-κB signaling^26^, indicating that NF-κB2/p100 is important for induction of non-canonical NF-κB signaling. However, in the absence of NF-κB2, RelB can also use the p50 protein from the canonical pathway as a dimer partner for transcriptional activity^34,64^. In this context, it was noteworthy that, while NF-κB activity in WT Tregs consisted primarily of RelA and cRel, *Nfkb2*−/− Tregs showed higher NF-κB activity mainly involving RelB. Interestingly, multiple myeloma cells with mutations that lead to complete degradation of NF-κB2/p100 have been reported to show a similar enhancement of TNF-alpha-mediated RelB:p50 NF-κB activity, mediating the expression of pro-survival genes^34^.

A recent report has also shown that a Treg-specific deletion of RelB, did not bring about any alteration in the peripheral Treg numbers or their suppressive function^60^. This indicates, consistent with our results, that RelB is dispensable for the regulation of peripheral Treg homeostasis. Additionally, our data further indicate that RelB activity also likely participates in the greater prominence of effector Tregs in the absence of *Nfkb2*, making it possible that RelB regulates the transcription of genes involved in eTreg generation in addition to those involved in Treg proliferation and survival.

NF-κB2/p100 in T cells has been reported to have a negative regulatory effect, through regulation of co-stimulation, on TCR activation-driven events such as proliferation^65^. Treg cells show substantial TCR-mediated tonic signalling *in vivo*^66^. It is therefore possible that *Nfkb2*−/− Tregs show such heightened TCR-induced signaling, contributing to enhanced activation and proliferation as well as effector conversion *in vivo*, including the formation of CD103+ Tregs. Similarly, while our study finds altered prominence of iTregs in the absence of *Nfkb2*, it must be kept in mind that our definition of the iTreg/nTreg distinction is based on the expression of Helios. As already noted^38^, this is a tentative identification at best. Further, Helios-expressing Tregs have been shown to be enriched in eTregs^67,68^.Thus, it is in fact possible that Helios expression and iTreg and eTreg prominence in the Treg compartment are also modified by the enhancement of TCR-mediated tonic signalling *in vivo*. In this context, cRel^25^ and RelA^69^ have also been implicated in regulating Helios expression, making it possible that removal of the negative regulatory effect of NF-κB2/p100 on either cRel or RelA activity contributes to the observed differences in iTreg to nTreg ratio among *Nfkb2*−/− Tregs. Finally, despite these caveats, our finding that deficiency of *Nfkb2* results in poor Treg induction *in vitro* from naïve conventional CD4 T cells confirms previous similar findings^48,70^, and is consistent with our *in vivo* observation of a reduction in the iTreg compartment among *Nfkb2*−/− Tregs.

Another recent study has used a Treg-specific deletion of *Nfkb2* and has reported findings similar to ours in terms of increased prominence of Tregs and of the effector phenotype amongst them^48^. This report also showed that, while Treg-specific *Nfkb2-*deficient mice did not develop any disease at a young age, from about one year of age they began showing immune inflammatory lesions associated with poor Treg suppressive capacity^48^. These features required RelB activity, since mice with Tregs lacking both *RelB* and *Nfkb2* showed normal Treg functionality and no immune inflammation^48^.

However, when we compared the suppressive functions of WT and *Nfkb2*−/− Tregs sorted from younger mice, either parental mice or bi-parental chimeras, we found no differences. We have not yet examined suppressive effects of Tregs from aged bi-parental chimeras. It remains an intriguing possibility that, as *Nfkb2*−/− Treg compartments age, with the enhanced activation and proliferation observed in these cells, suppressive activity declines perhaps as a result of induction of Th17 programming as suggested in the previous report^48^.

Despite being apparently functionally normal in young mice, the *Nfkb2*−/− Tregs in our study do show some altered characteristics reported previously as well, namely, reduced levels of CTLA-4 and enhanced GITR levels possibly related to heightened noncanonical NF-κB signaling^48,71^. It has been shown earlier that an absence of Nfkb2-p100 provokes enhanced RelB:p50 activity via the canonical NF-κB pathway in mouse embryonic fibroblasts, and that RelB:p50 induces a distinct set of genes, which are not activated by RelA:p50 and are associated with cell survival and cell division^72^. Our biochemical analyses revealed heightened nuclear activity of RelB:p50 in functionally normal *Nfkb2*−/− Tregs, while our global transcriptomic studies established an altered gene expression pattern in them favoring pro-survival and pro-proliferative functions. These analyses indicated that Nfkb2-p100 may also modulate Treg functions by tuning FOXP3 activity. Taken together, we argue that increased nuclear RelB:p50 activity in *Nfkb2*−/− Tregs, in the absence of Nfkb2-p100, may contribute to their higher proliferation and survival. It is therefore now attractive to hypothesize that, in the absence of *Nfkb2*, it is the persistence of unchecked RelB-NF-kappaB activity that over time leads to compromised Treg phenotype, and eventually, function. Thus, pathways mediating enhanced Treg activation, proliferation and survival may also in time lead to loss of function, a balance that may be maintained by NF-κB2. Our study highlights interesting interplay between non-canonical and canonical NF-κB pathway intermediates involving *Nfkb2*.

## Materials and Methods

### Mice

All mouse strains used in this study: C57BL/6, B6.TCRsß∂−/−, C57BL/6-Tg(UBC-GFP)30Scha/J (B6.GFP), B6.SJL-Ptprca Pepcb/BoyJ (B6.SJL), B6.SJLXC57BL/6 F1(CD45.1^+^CD45.2^+^) and *Nfkb2*(p100)−/−^73^ were obtained from The Jackson Laboratory (Bar Harbor, ME, USA) and were bred in the small animal facility of the National Institute of Immunology, New Delhi, India. All mice were used at 6–12 weeks of age and bi-parental chimeras were used 2–4 months post reconstitution. All mice were maintained and used in accordance with the guidelines and with the prior approval of the duly constituted ‘Institutional Animal Ethics Committee’ (IAEC) of the National Institute of Immunology. All methods were performed in accordance with relevant guidelines and regulations. All experimental protocols were approved by the Institutional Animal Ethics Committee (IAEC) authorised for this purpose.

### *Ex-vivo* cell isolation

Mice were euthanised by cervical dislocation and various organs were dissected. Erythrocyte lysis using Gey’s solution was used where necessary. Livers were perfused prior to preparation of single-cell suspensions, and a Percoll gradient centrifugation step was used to isolate leukocytes.

### Flow cytometry

Cells from various tissues were stained with combinations of the following antibodies to either identify NCD4 T cells, non-T cells (CD90^−^) and various Treg subsets, and to analyse expression of specific intracellular/extracellular markers on Tregs. The antibodies used were; CD90 (5E10), CD4 (RM4-5), CD8 (53–6.7), CD44 (IM7), CD62L (MEL-14), CD25 (PC61.5), CD45.1 (A20), CD45.2 (104), HELIOS(22-F6), FOXP3(FJK-16s), CD357(GITR) (DTA-1), CD103(2E7), CD152(CTLA-4) (UC10-4B9), CD28(CD28.2), TIGIT(MBSA43), CD279(PD-1) (J43), IRF4(3E4), CD304(Neuropilin-1) (3DS304M), GARP(YGIC86), CD122(IL-2Rβ)(5H4), CD95(Fas)(15A7), CD215(IL-15Rα)(DNT15Ra) and Egr2 (erongr2) (eBioscience, ThermoFisher Scientific, Carlsbad, CA). Surface marker staining was performed in staining buffer (phosphate-buffered saline (PBS) + 1% fetal bovine serum (FBS) + 0.05%NaN3) at 4 °C for 20 min followed by two washes with staining buffer. For intracellular proteins, cells were first fixed and permeabilised using Foxp3/Transcription Factor Staining Buffer Set (eBioscience, ThermoFisher Scientific, Carlsbad, CA) and then stained with appropriate combinations of antibodies. Sytox dead cell stain (MolecularProbes, ThermoFisher Scientific, Carlsbad, CA) was used to stain dead cells in order to assess cell viability. Fixable Violet (Invitrogen, ThermoFisher Scientific, Carlsbad, CA) was used to label dead cells before fixation and permeabilisation. Flow cytometric data collection was done (FACSVerse; BD Biosciences) and data analysed with FlowJo software (Treestar, Ashland, OR).

### Bi-parental bone marrow chimera generation

B6.GFP or C57BL/6 X B6.SJL F1s (CD45.1^+^CD45.2^+^) were subjected to a 10 Gy dose of gamma-radiation (Source: Co^60^, BARC, Mumbai). Five million bone marrow cells each from WT (B6.SJL: expressing CD45.1) and *Nfkb2*−/− (expressing CD45.2) mice were transferred intravenously into the gamma-irradiated mice and were allowed to reconstitute. After 8 weeks, reconstitution of both donors within the recipient was assessed in the peripheral blood of the recipients. Mice with >80% overall donor reconstitution were used for analysis.

### Cell sorting

*Ex-vivo* spleen cells were suspended in complete RPMI1640 medium (Biological Industries, Beit Haemek, Israel) supplemented with 10% FBS, 2 mM L-glutamine, 1.35 g/l sodium bicarbonate, 5 mM HEPES, 50 μM β-mercaptoethanol, 100 μg/ml streptomycin and 100 IU penicillin (Millipore Sigma, Merck KGaA, Germany) and stained with the relevant antibody combinations and incubated at 4 °C for 20 min. The cells were then washed, re-suspended at a density of 30 million cells/ml in complete RPMI and filtered through a 40-micron size sieve. This was followed by analysis and sorting (BD FACSAria III; BD Biosciences, CA). Sorted populations showed >90% purity.

### Treg suppression assay

WT and *Nfkb2*−/− Tregs (CD4+CD25+) were sorted from bi-parental bone marrow chimeras and NCD4 T cells were sorted from B6.GFP mice and labelled with CellTrace violet dye. For estimating suppression *in vitro*, labelled NCD4 T cells were cultured in the absence or presence of varying Treg numbers with aqueous phase anti-mouse CD3 (2 µg/ml) and gamma-irradiated (10 Gy) CD90^−^ splenocytes from C57BL/6 mice in complete RPMI. After 60 h of culture, the degree of proliferation of the live NCD4 T cells was determined by dye dilution to test the suppressive function of the respective Treg population. For *in vivo* suppression assays, labelled NCD4 T cells were transferred into B6.TCRβδ−/− mice either without Tregs or with purified WT or *Nfkb2*−/− Tregs from bi-parental bone marrow chimeras respectively at a ratio of 3:1 (NCD4:Tregs).Three days post-transfer, proliferation of the transferred NCD4 T cells was assessed in the spleens of the recipient mice by dye dilution.

### *In vitro* iTreg generation

Generation of iTreg cells was performed according to a protocol described previously^74^. WT and *Nfkb2*−/− NCD4 T cells were sorted from the mixed bone marrow chimeras, mixed in a 1:1 ratio (0.1 million cells of each) and cultured *in vitro* with plate-bound anti-CD3 (5 µg/ml) and anti-CD28 (5 µg/ml) in the presence of recombinant IL-2 (10 U/ml) and recombinant TGF-β1 (10 ng/ml) in complete RPMI. After 3–5 days of culture, cells were stained with fixable violet dye, fixed and permeabilised and then stained with anti-mouse CD45.1, CD45.2, CD4, CD25 and FOXP3 to identify the frequency of Tregs generated from WT and *Nfkb2*−/− NCD4 T cells.

### *In vivo* parking experiment

WT and *Nfkb2*−/− central and effector Tregs respectively were sorted from mixed bone marrow chimeras and 0.3 million cells of each type were mixed together equally and intravenously transferred into CD45.1^+^CD45.2^+^ F1 mice. After 3–10 days of parking *in vivo*, spleen cells of the recipients were harvested and stained with anti-mouse CD45.1, CD45.2, CD4, CD25, FOXP3, CD62L and Ki67. Tregs were identified as CD4^+^CD25^+^FOXP3^+^ cells and among them the transferred populations were identified as WT (CD45.1^+^) or *Nfkb2*−/− (CD45.2^+^) Tregs. The transition from central to effector Treg phenotype (or vice versa) was determined by analysing the expression of CD62L on the transferred Tregs. The frequency of proliferating cells among the transferred populations was determined by assessing frequency of Ki67^+^ cells.

### RNA-seq analysis

WT and *Nfkb2*−/− Tregs were isolated as described above from spleens of bi-parental bone marrow chimeras. Sorted cells were rested in complete RPMI at 37 °C for 2 h before being re-suspended in 1 ml TRIzol reagent (Thermofisher Scientific, CA) and stored at −80 °C. Three such biological repeats of WT and *Nfkb2*−/− Tregs were collected. RNA was isolated by treatment with chloroform, isopropanol and ethanol according to the RNA isolation protocol using TRIzol reagent as recommended. The RNA extracted was quantified using NanoDrop1000 (Thermofisher Scientific, CA). Unstranded RNAseq libraries were prepared using TruSeq RNA Sample Prep Kit v2 (Ilumina, San Diego) according to the kit protocol, and barcoded libraries were sequenced in Illumina HiSeq2500 (Macrogen, S. Korea). Fastq files generated by paired-end sequencing reactions were quality controlled using FastQC package^75^, followed by mapping to the mouse genome using HISAT2 tool^76^. The BAM file generated was used to count the reads mapped to each gene with htseq-count^77^. Read quality check, mapping and counting were done in galaxy public server^78^. The read counts generated were analysed for differential gene expression using DEseq2 package^79^. g:profiler^80^ was used for gene ontology analysis of upregulated genes. Genomic sequence and the annotation file was obtained from ENSEMBL^81^. For gene set enrichment analysis, the GSEA tool (Broad institute) was used with the collections H, C2 and C5^82^. Log2-fold changes for genes belonging to the significantly enriched sets related to proliferation and survival were used for heatmaps.

### Statistical analyses

Student’s ‘t’ test was used to calculate statistical significance of experimental data.

## Supplementary information

Supplementary information

## Data Availability

The RNA-seq data generated and analysed in the manuscript have been made available by submission to the Geo repository (accession number awaited).
