# Supplementary material for: Role of NF-kappaB2-p100 in regulatory T cell homeostasis and activation
Source: Sci Rep. 2019 Sep 25;9:13867. doi: 10.1038/s41598-019-50454-z (PMC6761191; doi:10.1038/s41598-019-50454-z)
Supplement: Supplementary file 1 — Supplementary information [file 41598_2019_50454_MOESM1_ESM.pdf]

Supplementary information

Role of NF-kappaB2-p100 in regulatory T cell homeostasis and activation

Atika Dhar, Meenakshi Chawla, Somdeb Chattopadhyay, Neelam Oswal, Danish Umar, Suman Gupta, Vineeta Bal, Satyajit Rath, Anna George, G. Aneeshkumar Arimbasseri, Soumen Basak

National Institute of Immunology, New Delhi, India

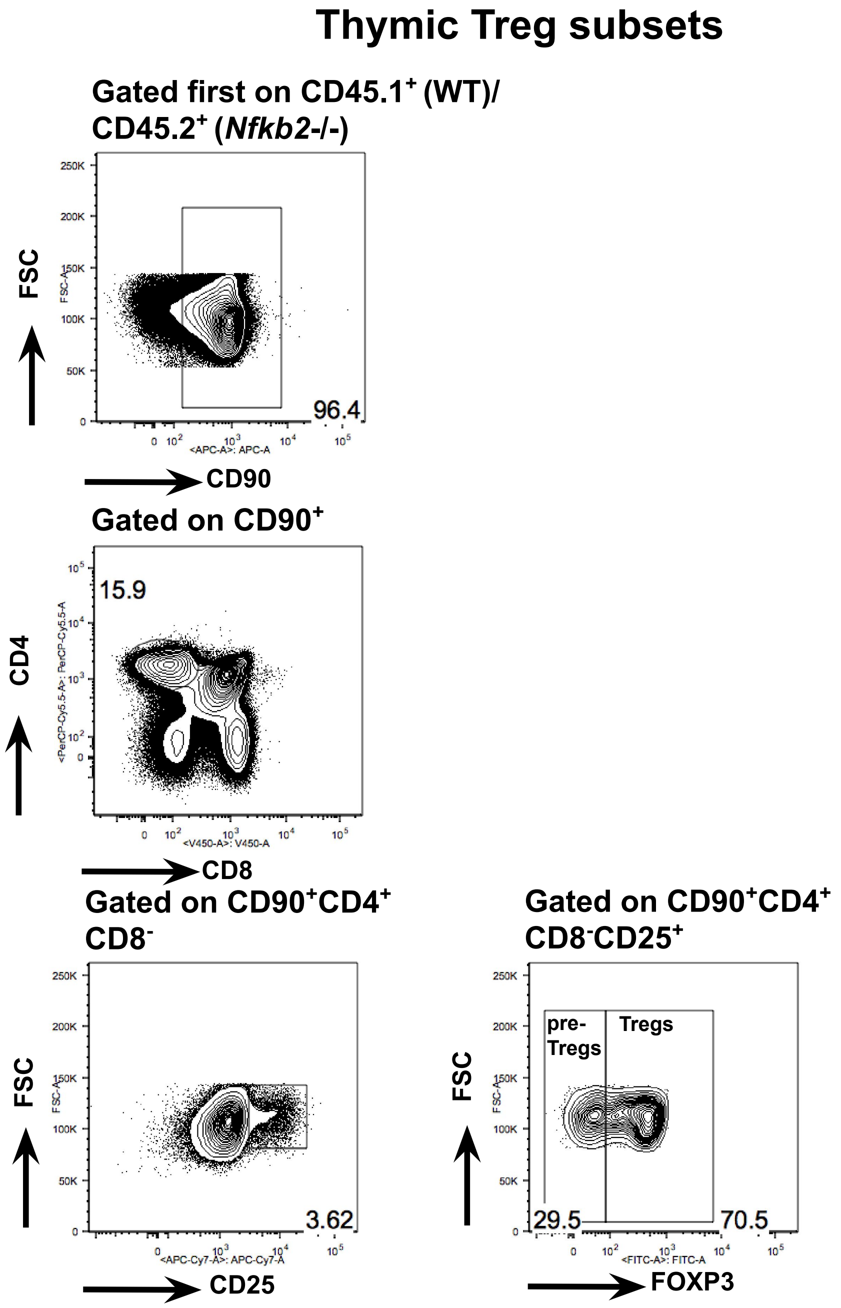

Fig. S1. *Gating strategy to identify Treg precursors and Tregs in the thymus*

Thymocytes were isolated from respective parental strains (WT/*Nfkb2*-/-) or from bi-parental bone marrow chimeras and stained with anti-mouse CD45.1, CD45.2, CD4, CD8, CD25 and FOXP3 to identify Treg precursors (pre-Tregs) ($CD4^{+}CD8^{-}CD25^{+}FOXP3^{-}$) and mature Tregs (Tregs) ($CD4^{+}CD8^{-}CD25^{+}FOXP3^{+}$), as described previously[36], both among WT ($CD45.1^{+}$) an *Nfkb2*-/- ($CD45.2^{+}$) mice strains/donors. CD4SP: CD4-single-positive thymocytes.

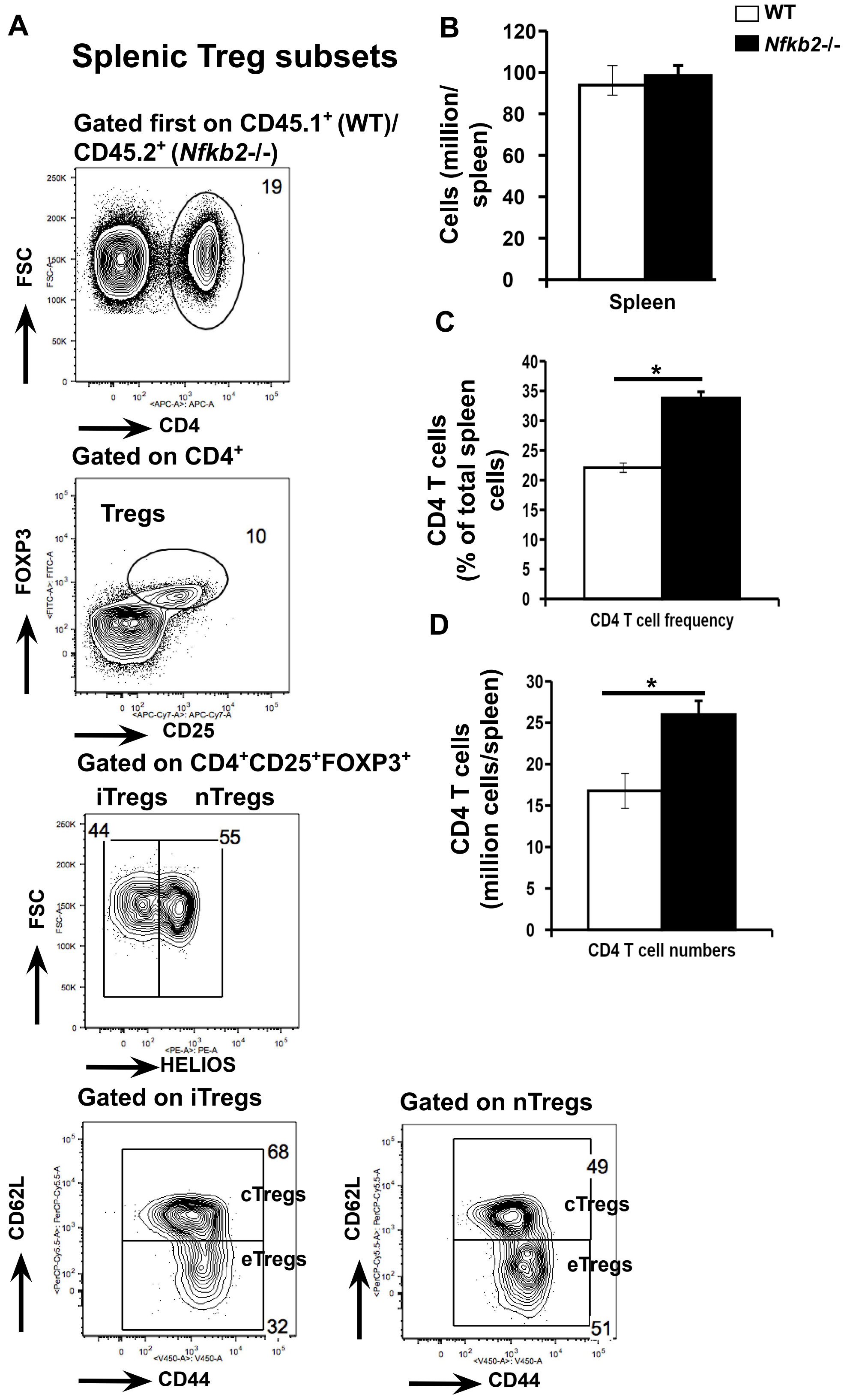

Fig. S2. Gating strategy to identify various peripheral Treg subsets

A: Cells were isolated from spleens of respective parent strains (WT/*Nfkb2*-/-) or from bi-parental bone marrow chimeras and stained with anti-mouse CD45.1, CD45.2,

CD4, CD25, FOXP3, HELIOS, CD62L and CD44 to identify various Treg subsets in the spleen, as described previously[14,37]. The following populations were thus identified: iTregs (induced Tregs)(CD4^{+}CD25^{+}FOXP3^{+}HELIOS^{low}), nTregs (natural Tregs) (CD4^{+}CD25^{+}FOXP3^{+}HELIOS^{high}) and among each of these subsets, cTregs (central Tregs): a) iTregs(CD4^{+}CD25^{+}FOXP3^{+}HELIOS^{low}CD62L^{high} CD44^{low}) and b) nTregs(CD4^{+}CD25^{+}FOXP3^{+}HELIOS^{high} CD62L^{high} CD44^{low}) and eTregs (effector Tregs): a) iTregs(CD4^{+}CD25^{+}FOXP3^{+}HELIOS^{low}CD62L^{low}CD44^{high}) and b) nTregs(CD4^{+}CD25^{+}FOXP3^{+}HELIOS^{high} CD62L^{low}CD44^{high}), both among WT(CD45.1^{+}) and *Nfkb2*-/- (CD45.2^{+}) mice strains/donors.

B-D: The bar graphs represent total spleen cell numbers (B), splenic CD4 T cell frequencies (C) and numbers(D) in WT and *Nfkb2*-/- mice respectively.

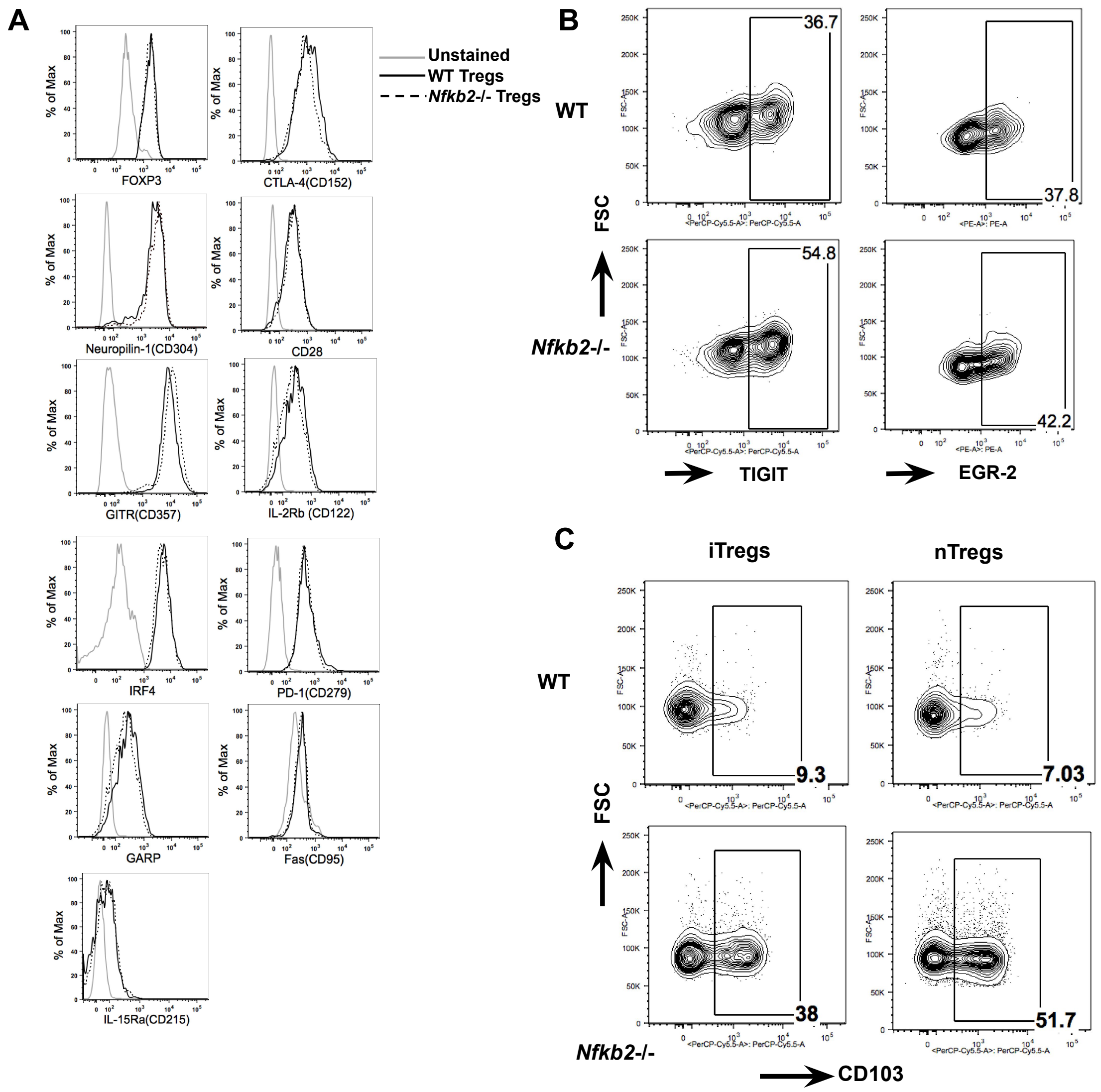

Fig S3. Qualitative analysis of WT and Nfkb2-/- Tregs

Spleen cells were isolated from WT and *Nfkb2*-/- bi-parental bone marrow chimeras and fixed, permeabilised and stained,

A: with anti-mouse CD45.1, CD45.2, CD4, CD25, FOXP3 and one of the following marker-specific antibodies to analyse the expression of various Treg-associated markers on WT and *Nfkb2*-/- Tregs: CTLA-4 (CD152), Neuropilin-1 (CD304), CD28, GITR (CD357), IL2-Rβ (CD122), IRF4, PD-1 (CD279), GARP, Fas and IL-15Rα. Grey histograms represent unstained control, solid black histograms represent expression of the indicated marker on WT Tregs, and dashed black histograms represent expression on *Nfkb2*-/- Tregs.

B: with anti-mouse CD45.1, CD45.2, CD4, CD25, FOXP3 and either TIGIT or Egr2 to analyse the expression of these markers on WT and *Nfkb2*-/- Tregs. As TIGIT and

Egr2 expression showed a bimodal distribution, the representative figures display the frequency of TIGIT^{+} and Egr2^{+} WT and *Nfkb2*-/- Tregs respectively.

C: with anti-mouse CD45.1, CD45.2, CD4, CD25, FOXP3, HELIOS and CD103 to analyse the expression CD103 on WT and *Nfkb2*-/- iTregs and nTregs respectively. The figure is a representative of the frequency of CD103 expressing WT and *Nfkb2*-/- iTregs and nTregs respectively.

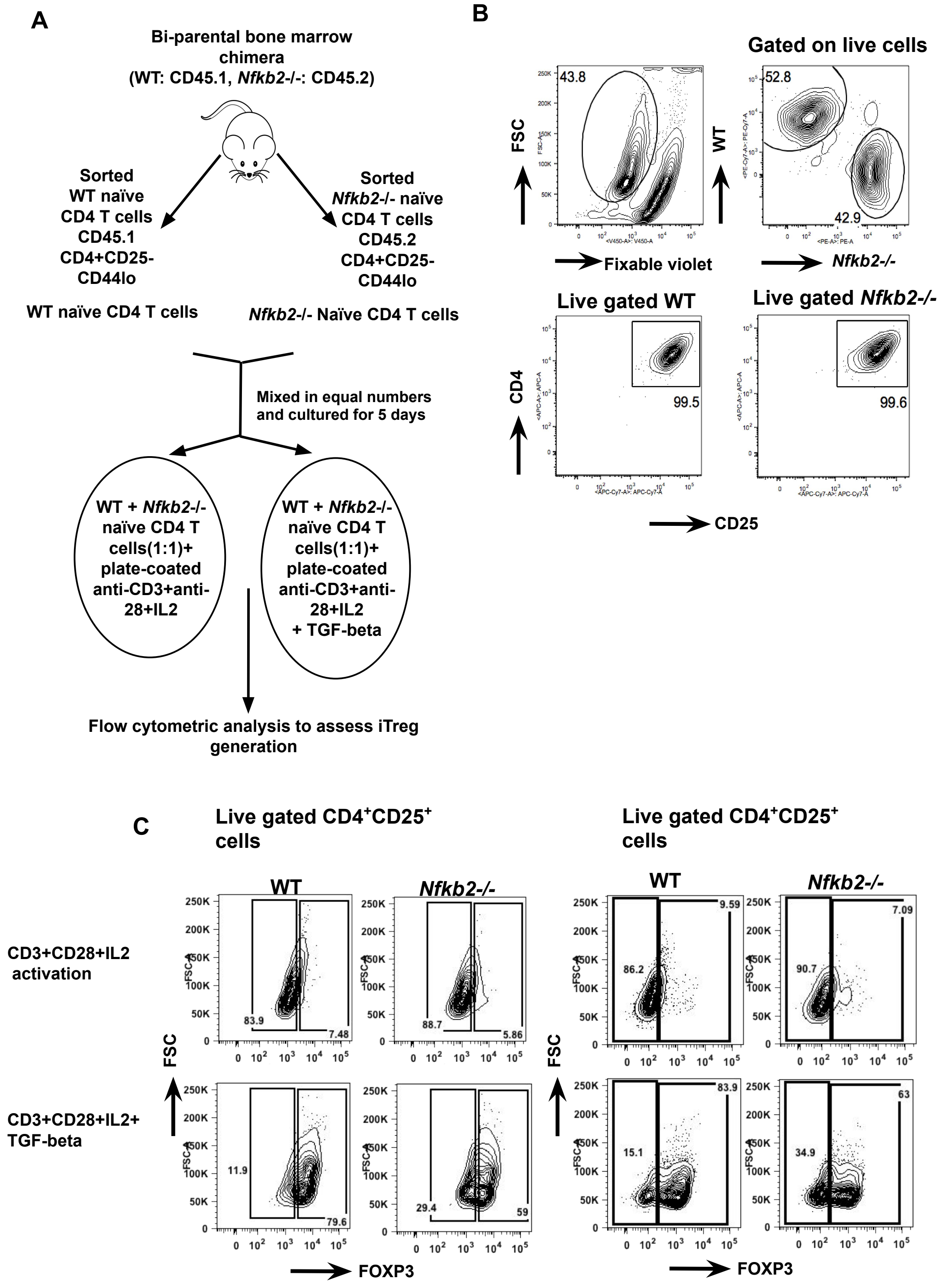

Fig. S4. Nfkb2-/- naïve CD4 T cells display poor in vitro Treg generation

A: Experimental schema for assay of in vitro iTreg induction. WT (CD45.1$^+$) and *Nfkb2*-/- (CD45.2$^+$) naïve CD4 (NCD4) T (CD4$^+$CD25$^-$CD44^{lo}) cells were sorted from bi-parental bone marrow chimeras, mixed 1:1 and stimulated in vitro in the presence of plate-bound anti-CD3+anti-CD28 (5 µg/ml), IL2 (10 U/ml) and either in absence or

presence of TGFβ1 (10 ng/ml) for five days, followed by flow cytometric analysis to assess iTreg induction by assessing FoxP3 expression.

B: After 5 days of activation as described in (A), live cells were identified on the basis of fixable violet dye exclusion and further distinguished as WT ($CD4^+CD25^+CD45.1^+$) and *Nfkb2*-/- ($CD4^+CD25^+CD45.2^+$) donor cells.

C: WT and *Nfkb2*-/- cells were assessed for FOXP3 expression to identify in vitro generated Tregs. Induction of FOXP3 expression was determined comparing conventionally activated CD4 T cells and iTregs (without and with TGFß respectively).

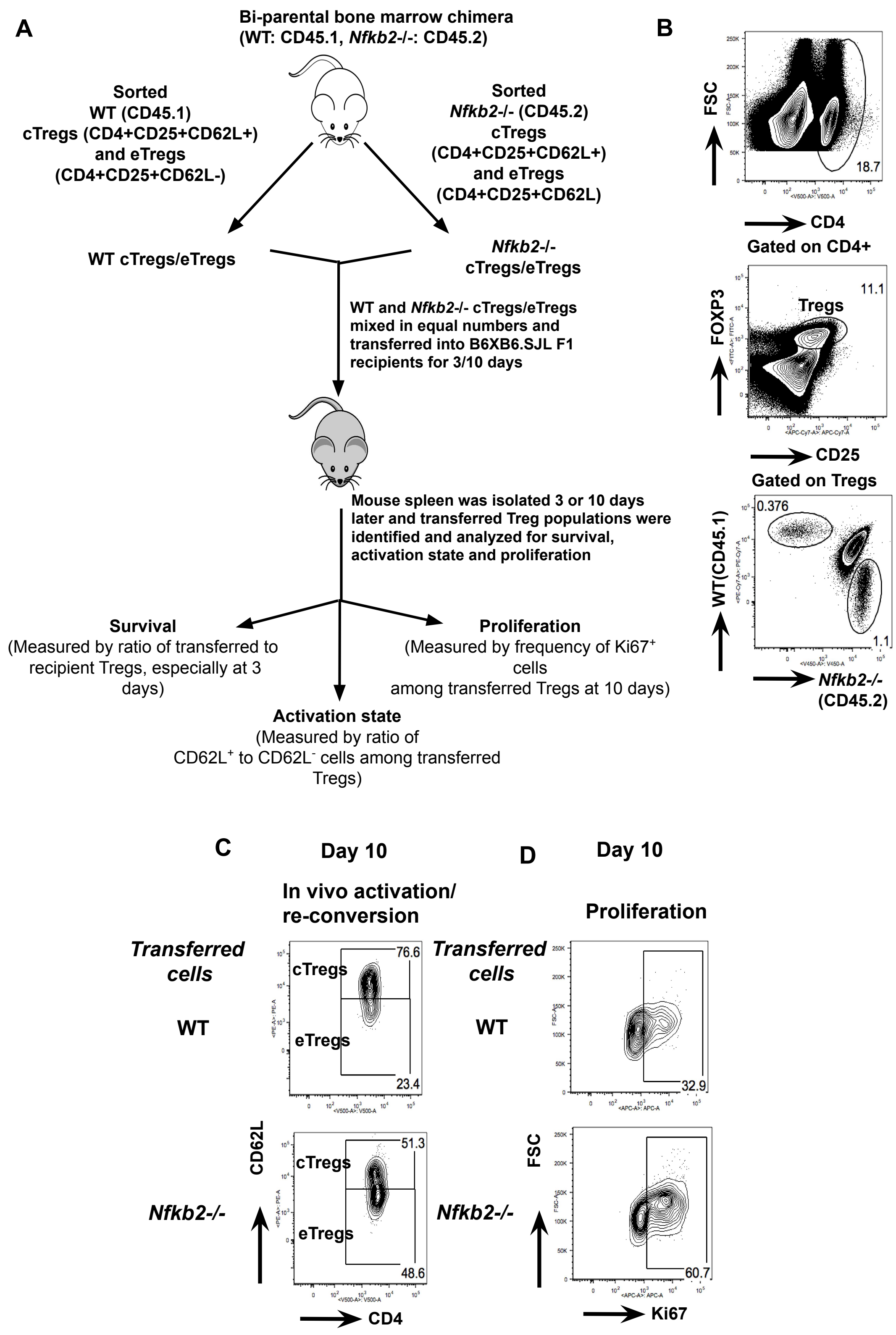

Fig. S5. In vivo parking of central and effector Tregs

A: Experimental schema for in vivo assays. Sort-purified splenic WT (CD45.1$^+$CD4$^+$CD25$^+$CD62L^{high}) and *Nfkb2*-/- (CD45.2$^+$CD4$^+$CD25$^+$CD62L^{high}) central or WT (CD45.1$^+$CD4$^+$CD25$^+$CD62L^{low}) and *Nfkb2*-/- (CD45.2$^+$CD4$^+$CD25$^+$CD62L^{low})

effector Tregs from bi-parental bone marrow chimeras were intravenously transferred in equal numbers into WT recipients ($CD45.1^+CD45.2^+$ F1 mice) for 3 or 10 days. Spleens were then harvested from the recipients and spleen cells were fixed, permeabilised and stained with anti-mouse CD45.1, CD45.2, CD4, CD25, FOXP3, CD62L and Ki67 to compare survival, activation and proliferation of transferred WT and *Nfkb2*-/- Treg populations.

B: Tregs were identified as $CD4^+CD25^+FOXP3^+$ cells and the transferred populations further gated on Tregs as WT ($CD45.1^+$) and *Nfkb2*-/- ($CD45.2^+$) Tregs respectively.

C: Conversion of central to effector Tregs (and vice-versa) after in vivo parking was assessed by calculating the ratio of cTregs ($CD62L^+$) to eTregs ($CD62L^-$).

D: Proliferation in the transferred Treg populations was assessed by the frequencies of $Ki67^+$ cells.

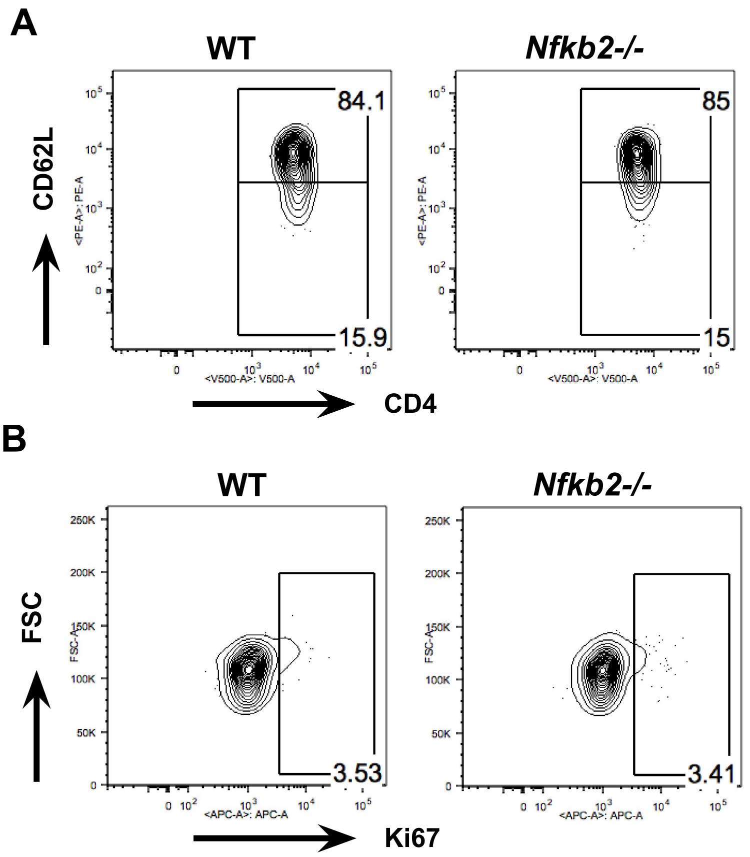

Fig. S6. Nfkb2-/- central Tregs show better survival in vivo

WT ($CD45.1^{+}CD4^{+}CD25^{+}CD62L^{high}$) and *Nfkb2*-/- ($CD45.2^{+}CD4^{+}CD25^{+}CD62L^{high}$) central Tregs respectively were sorted from the spleens of bi-parental bone marrow chimeras and transferred in equal numbers into $CD45.1^{+}CD45.2^{+}$ F1 mice for 3 days. Transferred populations were then identified in the spleens of the recipients to assess differences in their survival and also assessed for activation and proliferation. (A) CD62L expression and (B) frequencies of proliferating cells among transferred WT and *Nfkb2*-/- cTregs respectively were assessed at three days post transfer.

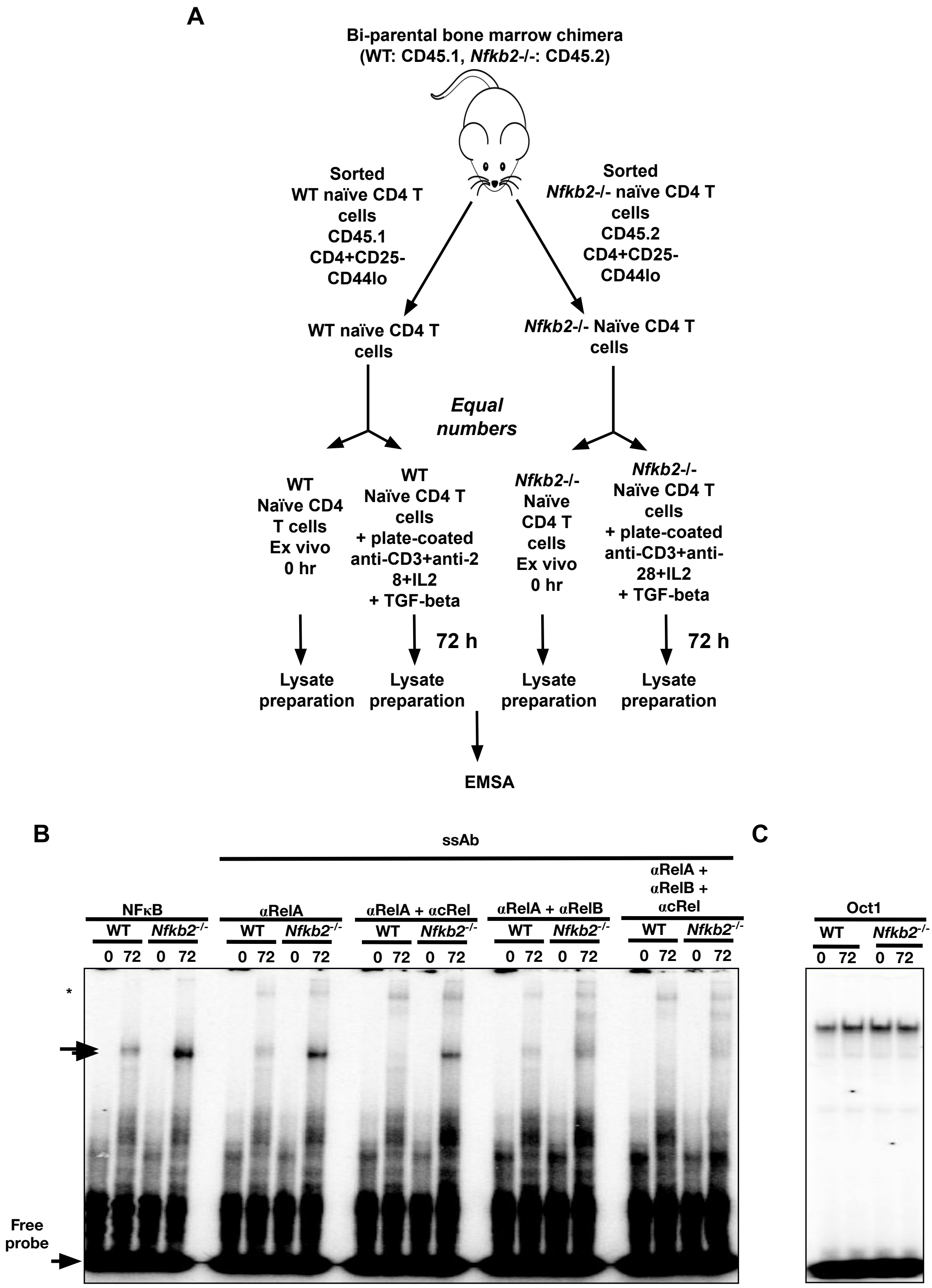

Fig. S7. In vitro generated Nfkb2-/- Tregs show higher NF-κB activity

Tregs were generated in vitro from WT and *Nfkb2*-/- NCD4 T cells under iTreg-inducing conditions using anti-CD3+anti-CD28+TGFβ+IL2, and subjected to an EMSA to assess nuclear NF-κB activity, as described in schema (A). Supershift assays were done with anti-RelA, anti-RelA+anti-cRel and anti-RelA+anti-RelB

antibodies to identify the relative prominence of RelA, RelB and cRel in these assays. The gel image depicts NF-κB activity and the relative prominence of RelA, RelB and cRel (left to right) (B) and the levels of Oct1 as a loading control (C) in WT and *Nfkb2*-/- NCD4s respectively at 0 h and post 72 h of culture under Treg inducing conditions in vitro. Arrow and arrowhead represent RelA-, RelB- and cRel- containing NF-κB activity, respectively. Asterisk represents supershifted complexes. ssAb: supershift antibody.

Table S1. Differential gene expression in Nfkb2-/- Tregs. Results of DEseq2 analysis of the RNAseq data.

https://github.com/aneeshkag/Atika_et_al
